# A Model-Based Analysis of GC-Biased Gene Conversion in the Human and Chimpanzee Genomes

**DOI:** 10.1371/journal.pgen.1003684

**Published:** 2013-08-15

**Authors:** John A. Capra, Melissa J. Hubisz, Dennis Kostka, Katherine S. Pollard, Adam Siepel

**Affiliations:** 1 Gladstone Institutes, University of California, San Francisco, California, United States of America; 2 Department of Biological Statistics and Computational Biology, Cornell University, Ithaca, New York, United States of America; 3 Department of Developmental Biology and Computational & Systems Biology, University of Pittsburgh, Pittsburgh, Pennsylvania, United States of America; 4 Institute for Human Genetics and Division of Biostatistics, University of California, San Francisco, California, United States of America; University of California Davis, United States of America

## Abstract

GC-biased gene conversion (gBGC) is a recombination-associated process that favors the fixation of G/C alleles over A/T alleles. In mammals, gBGC is hypothesized to contribute to variation in GC content, rapidly evolving sequences, and the fixation of deleterious mutations, but its prevalence and general functional consequences remain poorly understood. gBGC is difficult to incorporate into models of molecular evolution and so far has primarily been studied using summary statistics from genomic comparisons. Here, we introduce a new probabilistic model that captures the joint effects of natural selection and gBGC on nucleotide substitution patterns, while allowing for correlations along the genome in these effects. We implemented our model in a computer program, called phastBias, that can accurately detect gBGC tracts about 1 kilobase or longer in simulated sequence alignments. When applied to real primate genome sequences, phastBias predicts gBGC tracts that cover roughly 0.3% of the human and chimpanzee genomes and account for 1.2% of human-chimpanzee nucleotide differences. These tracts fall in clusters, particularly in subtelomeric regions; they are enriched for recombination hotspots and fast-evolving sequences; and they display an ongoing fixation preference for G and C alleles. They are also significantly enriched for disease-associated polymorphisms, suggesting that they contribute to the fixation of deleterious alleles. The gBGC tracts provide a unique window into historical recombination processes along the human and chimpanzee lineages. They supply additional evidence of long-term conservation of megabase-scale recombination rates accompanied by rapid turnover of hotspots. Together, these findings shed new light on the evolutionary, functional, and disease implications of gBGC. The phastBias program and our predicted tracts are freely available.

## Author Summary

Interpreting patterns of DNA sequence variation in the genomes of closely related species is critically important for understanding the causes and functional effects of nucleotide substitutions. Classical models describe patterns of substitution in terms of the fundamental forces of mutation, recombination, neutral drift, and natural selection. However, an entirely separate force, called GC-biased gene conversion (gBGC), also appears to have an important influence on substitution patterns in many species. gBGC is a recombination-associated evolutionary process that favors the fixation of strong (G/C) over weak (A/T) alleles. In mammals, gBGC is thought to promote variation in GC content, rapidly evolving sequences, and the fixation of deleterious mutations. However, its genome-wide influence remains poorly understood, in part because, it is difficult to incorporate gBGC into statistical models of evolution. In this paper, we describe a new evolutionary model that jointly describes the effects of selection and gBGC and apply it to the human and chimpanzee genomes. Our genome-wide predictions of gBGC tracts indicate that gBGC has been an important force in recent human evolution. Our publicly available computer program, called phastBias, and our genome-wide predictions will enable other researchers to consider gBGC in their analyses.

## Introduction

Gene conversion is the nonreciprocal exchange of genetic information from a ‘donor’ to an ‘acceptor’ sequence, primarily resulting from the repair of mismatched bases in heteroduplex recombination intermediates during meiosis [1]. In many cases, the process of resolving mismatches between G/C (guanine or cytosine; denoted ‘strong’ or ‘S’) and A/T (adenine and thymine; ‘weak’ or ‘W’) alleles appears to be biased in favor of S alleles [1]–[3]. Such GC-biased gene conversion (gBGC) elevates the fixation probabilities for S alleles relative to W alleles at positions of W/S polymorphism, and, if it acts in a recurrent manner over a sufficiently long time, can result in a significant excess of W→S over S→W substitutions and a consequent increase in equilibrium GC content. It has been known since the 1980s both that gene conversion occurs in various eukaryotes [4] and that mismatch repair can be significantly biased [5]. As complete genome sequences have become widely available, evidence has accumulated that gBGC may have played an important role in genomic evolution across many branches of the tree of life. In particular, it has been argued that gBGC has significantly influenced the genomic distribution of GC content, the fixation of deleterious mutations, and rapidly evolving sequences in many species [6]–[13].

Aside from limited experimental evidence of a GC-bias in meiosis, mostly from yeast [14], much of what is known about gBGC comes from two indirect sources of information: global patterns of variation within or between species suggesting a fixation bias favoring S alleles [11], [12], [15]–[17] and the existence of numerous loci exhibiting dense clusters of substitutions with a pronounced W→S bias [7]–[9], [13]. Both types of evidence correlate strongly with recombination rates, consistent with the hypothesis that they are caused by gBGC, although other recombination-associated factors might also contribute [16]. However, these observations provide limited information about the general prevalence, strength, and functional consequences of gBGC in humans and other mammals. Genome-wide patterns of variation are influenced by diverse forces that act in a highly heterogeneous manner across the genome, and it is difficult to measure the specific contribution of gBGC to these patterns. Clusters of biased substitutions perhaps provide more direct evidence of a local influence from gBGC. However, such clusters have so far been identified by considering either genomic windows of fixed size or pre-identified genomic segments (such as protein-coding exons or fast-evolving noncoding regions), which has limited the regions that can be detected. In addition, many studies have considered only fairly small numbers of clusters showing extreme substitution rates and W→S biases.

For modelers of molecular evolution, gBGC is an anomaly—a process separate and distinct from the fundamental processes of mutation, recombination, drift, and selection that underlie most models, yet one with the potential to profoundly influence patterns of variation within and between species. Like selection, gBGC acts in the window between the emergence of genetic polymorphism due to mutation and its elimination due to the fixation or loss of derived alleles. Unlike selection, however, gBGC is neutral with respect to fitness. The influence of gBGC at individual nucleotides can be modeled approximately by treating it as a selection-like force that depends only on whether a new mutation is W→S, S→W, or neither [13], [16], [18]. However, this approach ignores the close association of gBGC with the notoriously difficult-to-model process of recombination, which leads to a complex correlation structure along the genome (i.e., gBGC “tracts” separated by regions of no gBGC). Owing to these difficulties, with a few exceptions [9], [13], [19], gBGC has generally been ignored in phylogenetic or population genetic models, and considered at most in post hoc analyses (e.g., by examining identified genomic regions for an excess of W→S substitutions). These approaches are clearly limited in efficiency and effectiveness, and there is a need for improved models of gBGC that can be applied on a genome-wide scale. There is also a need for high quality annotations of gBGC-affected regions that can be used by investigators in other comparative and population genomic analyses.

Another reason to develop improved models of gBGC is that gBGC-induced nucleotide substitutions provide a unique window into historical recombination processes, by serving as a proxy for average recombination rates along a lineage of interest. By contrast, the other main sources of information about recombination—sperm typing [20], genotypes for known pedigrees [21], and patterns of linkage disequilibrium in present-day populations [22]—provide information about recombination that goes back no farther than the coalescence time between individuals. Pronounced differences between the human and chimpanzee recombination maps suggest that recombination rates in hominoids have changed rapidly [23]–[25]. gBGC may provide useful information about the recombination processes during the critical period between the divergence of humans and chimpanzees (4–6 million years ago [Mya]) and the coalescence time for human individuals (roughly 1 Mya, on average). Notably, archaic hominin genome sequences are of limited use for this purpose, because they are still few in number and result in only a modest increase in coalescence times.

In this article, we address these issues by introducing a novel model-based approach for the identification of gBGC tracts. Our approach makes use of statistical phylogenetic models that jointly consider gBGC and natural selection [13]. In addition, it approximates the recombination-associated correlation structure of gBGC along the genome using a hidden Markov model. We have implemented this approach in a computer program called phastBias, which is available as part of the open-source PHylogenetic Analysis with Space/Time models (PHAST) software package (http://compgen.bscb.cornell.edu/phast) [26]. Using simulations, we show that phastBias can identify tracts of various lengths from unannotated multiple alignments with good power. We then analyze genome-wide predictions of gBGC tracts in the human and chimpanzee genomes, comparing them with recombination rates, patterns of polymorphism, functional elements, fast-evolving sequences, and other genomic features. This analysis sheds light on the prevalence and fitness consequences of gBGC, and on recombination processes during the time since the human/chimpanzee divergence. Our predictions of gBGC tracts are freely available as browser tracks (http://genome-mirror.bscb.cornell.edu). We anticipate that these tracks will be useful for avoiding false positives in scans for positive selection, understanding the evolution of specific loci, and investigating the broader evolutionary forces shaping the human genome.

## Results

### Probabilistic Model

**Figure 1:**
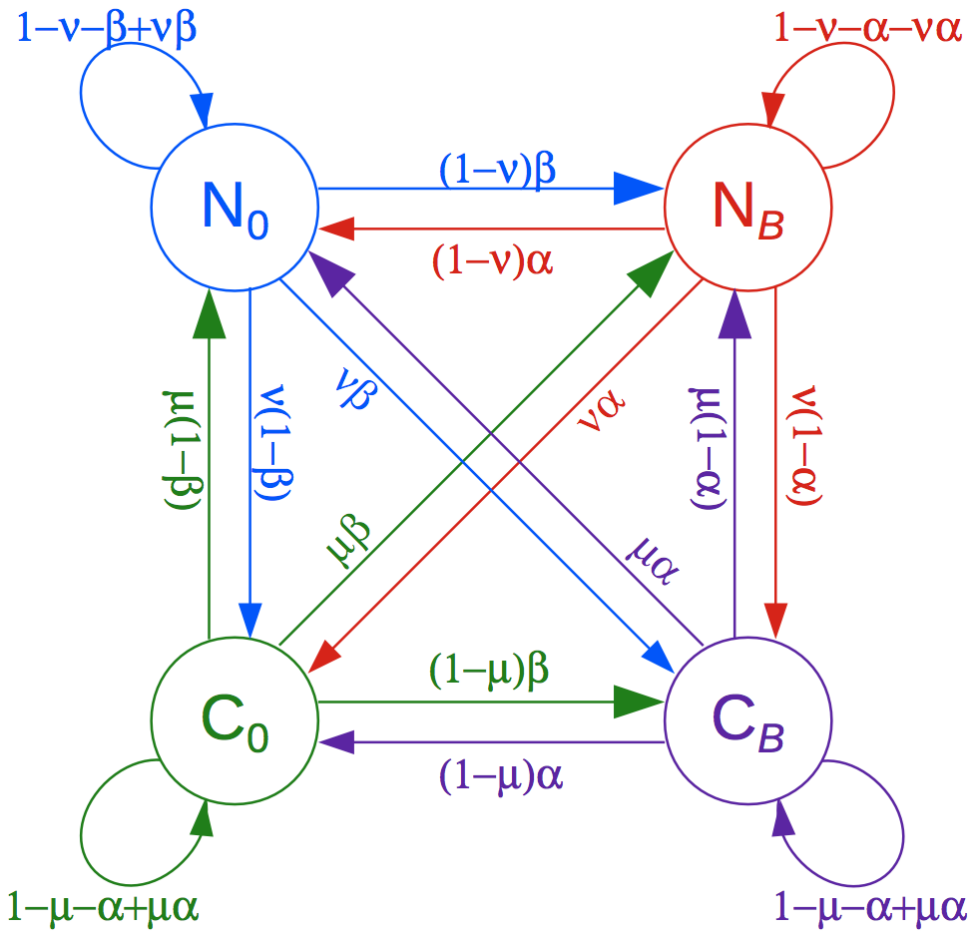
Phylogenetic hidden Markov model used by phastBias. The model consists of four states: neutral evolution with no gBGC (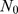), neutral evolution with gBGC (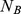), evolutionary conservation with no gBGC (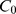), and evolutionary conservation with gBGC (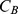). gBGC is assumed to influence nucleotide substitution rates and patterns only on the lineage leading to a designated target genome (human or chimpanzee in this study). The model generalizes the phylo-HMM used by phastCons for prediction of evolutionarily conserved elements [28]. The state transition probabilities are defined by four parameters, denoted 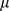, 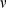, 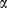, and 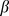. See Methods and Table 1 for details.

We model gBGC tracts using a phylogenetic hidden Markov model (phylo-HMM) with four states, representing all combinations of gBGC or no gBGC in a specified “target” genome (e.g., human or chimpanzee), and of evolutionary conservation or no evolutionary conservation across the phylogeny (Figure 1; Methods). The phylo-HMM framework [27] allows the distinct rates and patterns of nucleotide substitution for each state to be described using a full statistical phylogenetic model, and it captures the pronounced correlations along the genomes in these patterns using a first-order Markov model. Our phylo-HMM can be thought of as a straightforward generalization of the two-state model used by the phastCons program for prediction of evolutionarily conserved elements [28] that additionally predicts gBGC tracts in the target genome. We directly consider evolutionary conservation together with gBGC because the dramatic reduction in substitution rates in functional elements would otherwise be a confounding factor in the identification of gBGC tracts. The model allows conserved elements and gBGC tracts to overlap or occur separately. The joint effects of gBGC and selection are modeled by treating gBGC as a selection-like force that specifically favors the fixation of G and C alleles, as in other recent work. In particular, the influence of selection is described using a population-scaled selection coefficient, 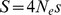, and the influence of gBGC is described using an analogous population-scaled GC-disparity parameter, 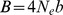 (where 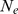 is the effective population size) [13] (see also [16], [18]). The parameter 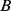 measures the strength of gBGC, and values 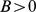 cause W→S substitution rates to increase and S→W substitution rates to decrease. A key feature of our approach is that it permits identification of gBGC tracts of any length based on characteristic substitution patterns, independent of predefined windows or genomic annotations.

Because the signal for gBGC in the data is typically quite weak, we make several assumptions to reduce the complexity of the model. Briefly, we model negative selection as uniformly decreasing evolutionary rates on all lineages, we ignore positive selection, and we assume that the disparity parameter 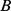 is the same for all gBGC tracts. In addition, we pre-estimate the parameters describing the neutral phylogeny and evolutionary conserved elements using restricted models, we fix the tract-length parameter 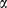 based on our prior expectation for tract lengths, and we treat the parameter 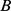 as a “tuning” parameter to be set by trial and error (see summary of model parameters in Table 1). Our simulation study indicates that fairly high accuracy in tract prediction is possible despite these simplifying assumptions and approximations (see below and Methods for details). We have implemented our model in a program called phastBias in the PHAST package [26]. PhastBias makes use of existing features in PHAST for alignment processing, phylogenetic modeling, efficient HMM-based inference, and browser track generation.

**Table 1:** Summary of HMM parameters.

Parameter	Group^a^	Description	Value
*λ*	neut	Scale factor for neutral branch lengths	estimated per 10 Mb block
*π* b	neut	Equilibrium nucleotide frequencies	estimated per 10 Mb block
*κ*	neut	Transition/transversion ratio	estimated per 10 Mb block
*ρ*	cons	Branch length scale factor in conserved state	0.31^c^
*μ*	cons	Transition prob. conserved→neutral	0.022^c^
*ν*	cons	Transition prob. neutral→conserved	0.0095^c^
*B*	gBGC	GC-disparity (strength of gBGC)	2, 3^d^, 4, 5, 10
*α*	gBGC	Transition prob. gBGC→non-gBGC	0.001^e^
*β*	gBGC	Transition prob. non-gBGC→gBGC	optimized by EM

a neut = parameters for neutral phylogenetic model, cons = parameters for conserved elements (inherited from phastCons), gBGC = parameters for gBGC tracts.

b Multivariate parameter (three degrees of freedom).

c Values used for the Conservation tracks in the UCSC Genome Browser (see [28]).

d Value used for primary analyses.

e Corresponds to prior expected length of 1 kb.

### Simulation Study

While the absence of high-quality annotations of gBGC tracts makes it difficult to assess prediction accuracy, we are able to gain some insight into the performance of phastBias using simulated data. To make our simulated data as realistic as possible, we started with real genome-wide alignments, and simulated new human sequences only, using our phylogenetic model to define neutral and conserved sequences, and interspersed gBGC tracts of fixed lengths (see Methods). This strategy ensures that features such as variation in mutation rates, changes in equilibrium GC content, conserved elements, indels, alignment errors, and missing data are all retained in the nonhuman sequences. We used phastBias to predict human-specific tracts based on these partially simulated alignments and compared our predictions with the “true” tracts assumed during simulation. We found that the nucleotide-level false positive rate was always very low in these experiments (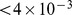/bp, Figure S1), so we measured the specificity of our predictions using the nucleotide-level positive predictive value (PPV), defined as the fraction of all bases predicted to be in gBGC tracts that truly belong in gBGC tracts. As a measure of power, we used the nucleotide-level true positive rate (TPR), the fraction of bases in true gBGC tracts that were correctly predicted as being in tracts.

**Figure 2:**
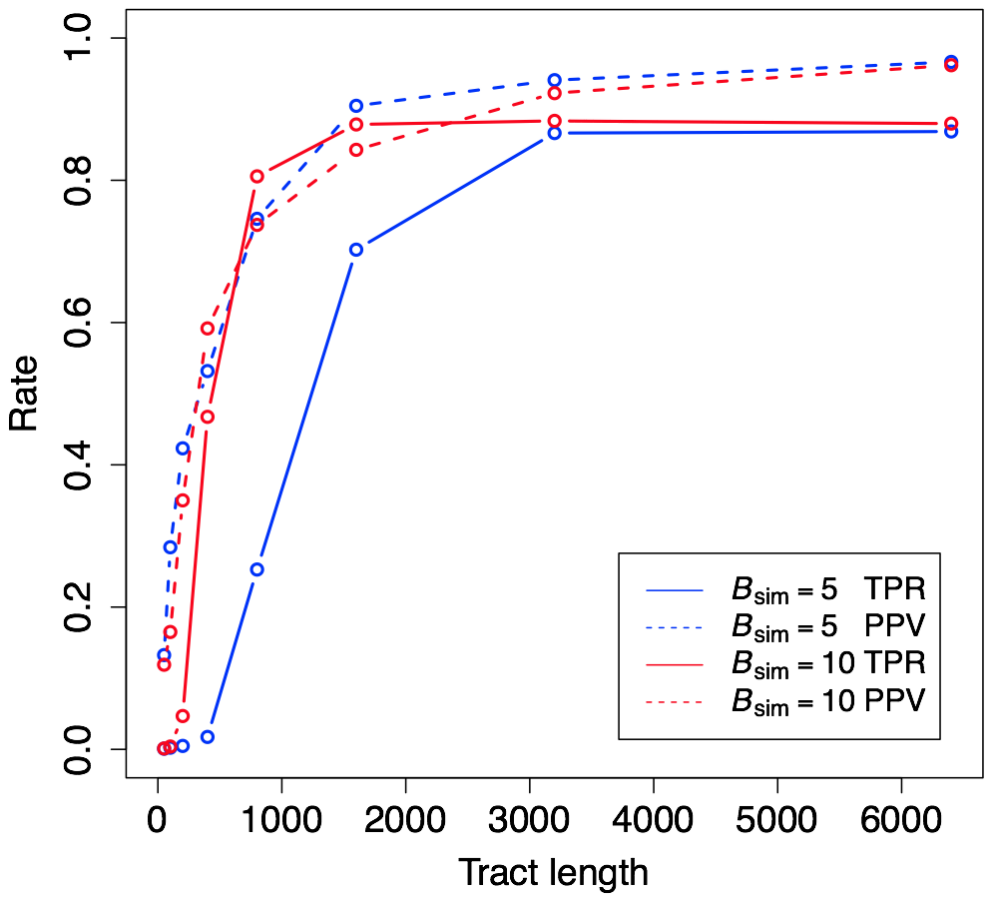
Power and accuracy for simulated data. The plot shows true positive rates (TPR; fraction of true gBGC bases correctly predicted) and positive predictive values (PPV; fraction of predicted bases in true gBGC tracts) as a function of tract length. Results are shown for two sets of simulations, one assuming strong BGC (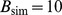), and the other assuming weaker BGC (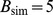) (see Methods). Both the power (as measured by the TPR) and the accuracy (as measured by PPV) of gBGC detection depend strongly on tract length. At shorter lengths (less than 3000 bp) power also depends strongly on the strength of gBGC, while accuracy does not. Both TPR and PPV are fairly high (80% or more) for tracts longer than 1 kb that have experienced strong gBGC, and for tracts longer than 1.6 kb that have experienced weaker gBGC.

First, we explored the performance of phastBias on simulated gBGC tracts of various lengths, generated with several different values of the GC-disparity parameter (denoted 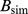). Under our model, increasing 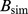 produces tracts with more substitutions and greater GC bias in their substitution patterns. As expected, both our power to detect gBGC and the specificity of our predictions increases with the lengths of the true tracts and with 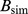 (Figure 2). We found that power and specificity were both quite good for tracts of 1,000–1,500 bases or longer, provided gBGC is reasonably strong (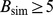). Current estimates of the lengths and GC-disparity of real gBGC tracts [8], [29] suggest that phastBias should have good power for many tracts (see Discussion).

Next, we examined how our choice of the tuning parameters for expected tract-length (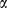) and gBGC strength (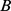) influence prediction performance. We found that the performance of the method was not highly sensitive to the value of 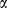, so we decided to fix the expected tract length at 1 kilobase (kb) (by setting 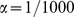) based on empirical evidence indicating that mammalian gene conversion tracts are approximately this size [1], [29]. By contrast, the choice of 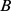 had a much stronger influence on the observed prediction performance. Power was highest for small values of 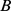, regardless of the value used to simulate the tracts (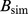) (Figure S2). However, this increase in power comes at only a modest cost in PPV, which remains fairly high (>90%) except when the elements are both short and under weak gBGC (e.g., mean length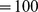 bases, 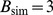). These results suggest that phastBias is inherently somewhat conservative with its predictions, and that setting 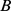 to a relatively low value helps to improve sensitivity for tracts having a range of true gBGC intensities, at minimal cost in specificity.

### Predicted gBGC Tracts

We applied phastBias to genome-wide alignments of the human, chimpanzee, orangutan, and rhesus macaque genomes, and used it to predict tracts in the human and chimpanzee genomes likely to have experienced gBGC since the divergence of these two species 4–6 Mya (see Methods). In separate runs, we selected either the human or the chimpanzee genome as the “target,” and we set the tuning parameter 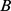 to values of 2, 3, 4, 5, and 10 (in increasing strength of gBGC). As expected from our simulation study, the number, lengths, and genomic coverage of the predicted tracts depend fairly strongly on the choice of 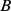. In particular, coverage decreases from more than 1% to 0.07% as 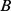 is increased from 2 to 10 (Table 2). Because the tracts predicted with high 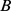 are largely found within those predicted with lower 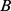 (Table S1), and because a value of 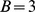 appears to result in good power while controlling false positives (see above), we will focus on the tracts predicted with 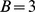 for the remainder of the article. The absolute sensitivity of these predictions of course depends on unknown properties of true gBGC tracts, but our simulation experiments indicate that power is fairly good, at least for the subset of tracts 1 kb or longer with a reasonably pronounced GC-disparity (Figure 2).

**Table 2:** Summary of predicted gBGC tracts.

Species	B	Number	Coverage	Mean Length	Median Length
Human	2	12362	1.103%	2567	1008
Human	3	9439	0.334%	1018	788
Human	4	7712	0.217%	810	628
Human	5	6750	0.157%	670	514
Human	10	5210	0.073%	400	276
Chimpanzee	3	8677	0.252%	841	663
Chimpanzee	10	7062	0.068%	278	198

**Figure 3:**
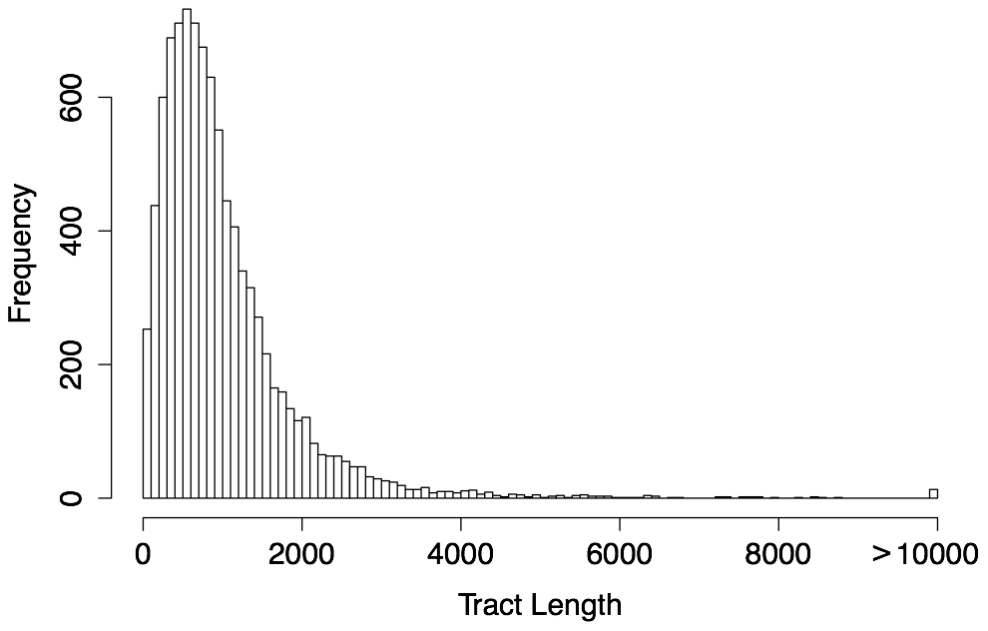
Length distribution of predicted human gBGC tracts (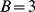). The predicted tracts average 1,018 bp in length, with a median value of 788 bp. The length distribution is roughly geometric except for a deficiency of short tracts (less than 600 bp) and a slight excess of long tracts. The deficiency of short tracts is typical for predictions based on a hidden Markov model and most likely primarily reflects limitations of power in this range. Nevertheless, the full distribution suggests that phastBias can identify tracts ranging from a few hundred to several thousand bases in length.

With 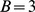, the predictions for the human genome include 9,439 gBGC tracts covering 0.33% of the genome (Table 2). These predicted tracts average 1,018 bp in length (median 788 bp), consistent with our choice of 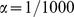, but they display a fairly broad length distribution (Figure 3), indicating that our choice of tuning parameters is not overly restrictive. Most predicted tracts contain exclusively or predominantly W→S substitutions (Figure S3). The statistics for the chimpanzee genome are similar, but in this case there are somewhat fewer tracts (8,677), their lengths are reduced (mean = 842 bp, median = 663 bp), and genomic coverage is about 25% lower (at 0.25%). The reduced coverage of the chimpanzee genome holds even if we consider only tracts that completely fall within regions of high-quality, syntenic alignment between the two genome assemblies. These differences between the human and chimpanzee predictions could reflect differences between species in the degree to which recombination events are concentrated in recombination hotspots [25] (see Discussion).

**Figure 4:**
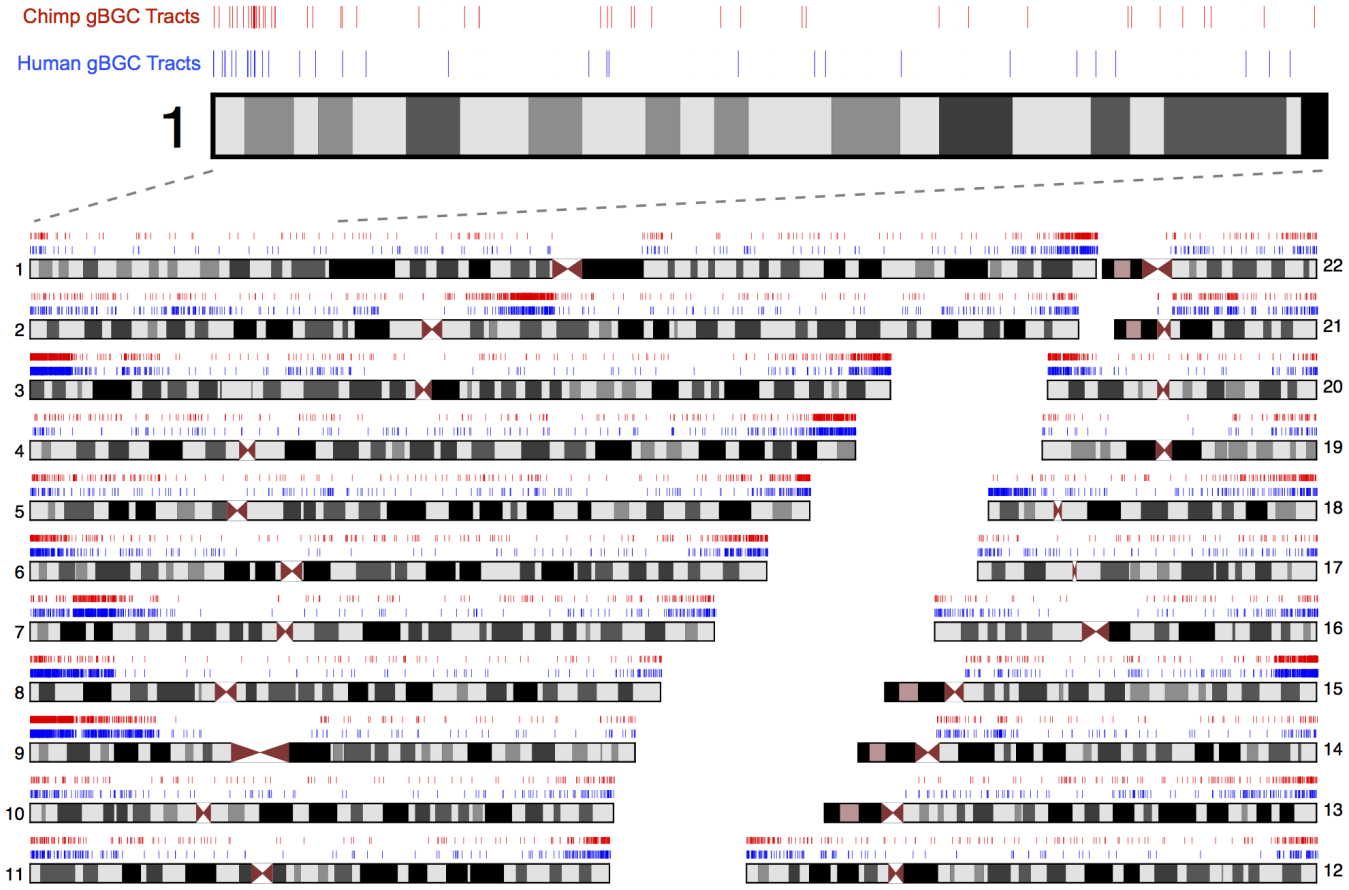
Genomic distribution of predicted human and chimpanzee gBGC tracts. Both human (blue) and chimpanzee (red) gBGC tracts are found throughout the genome, but tend to cluster and fall near telomeres. Chimpanzee gBGC tracts are displayed at the corresponding aligned positions in the human genome. The dense cluster of gBGC tracts near the centromere of chromosome 2 is the site of the fusion of two ancestral chromosomes on the human lineage. This region is telomeric in chimpanzee and was telomeric for much of human evolution. As illustrated by the magnified section of chromosome 1, human and chimpanzee tracts often occur in similar regions, but rarely overlap.

The human and chimpanzee predictions are broadly distributed across the two genomes, but show a clear tendency to cluster near the ends of chromosomes (Figure 4; Text S1, Figures S4 and S5), consistent with previous findings [12], [15], [30]. In human, the median distance from the nearest telomere is only about one third that observed for a set of GC-content-matched control regions (9.6 megabases (Mb) vs. an average of 30.4 Mb over 1000 replicates, 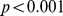). Similarly, the median distance between tracts is less than one third that for the controls, even after merging tracts less than 1 kb apart to account for possible biases from the HMM-based prediction method (24.3 kb vs. an average of 86.0 kb, 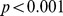). The chimpanzee predictions are similarly distributed. In human, there is an obvious cluster of predicted tracts near the centromere of chromosome 2, reflecting the telomeres of two ancestral chromosomes that fused at this site along the human lineage after the human/chimpanzee divergence [15], [31]. However, the tract density in this region is somewhat lower in human than in the orthologous telomeric regions in chimpanzee (Figure S6), consistent with a reduction in the human recombination rate following the fusion event [12], [15] (see Discussion).

Together, the human and chimpanzee tracts account for about 1.2% of all human/chimpanzee nucleotide differences apparent in our genome-wide alignments (435,729 differences). About half (214,195) of the nucleotide differences within the tracts can be confidently explained by W→S substitutions on either the human or chimpanzee lineage, of which slightly more than half (115,699) fall on the human lineage. Thus, even with our limitations in power, our predictions suggest a non-negligible influence of gBGC on overall levels of human/chimpanzee nucleotide divergence.

### Recombination Rates

The predicted human gBGC tracts are substantially enriched for recombination hotspots from the HapMap project [32]: 1,228 (13%) overlap a hotspot, compared with an average of 796 for the GC-matched control regions (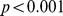). In addition, the average recombination rate [33] within these tracts is more than twice the rate in the control regions (3.85 centimorgans per megabase (cM/Mb) vs. 1.61 cM/Mb, 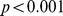; Table 3). A parallel analysis of the chimpanzee gBGC tracts based on the genome-wide recombination rate map from the PanMap Project [25] showed, similarly, that recombination rates in predicted gBGC tracts were more than twice as high as in control regions (Table 3). Pedigree-based human recombination maps [21] produced similar results (data not shown).

**Table 3:** Recombination rates in gBGC tracts.

Recombination Map	Human gBGC Tract Rate (cM/Mb)	Chimpanzee gBGC Tract Rate (cM/Mb)	GC-matched Control Rate (cM/Mb)
Human	3.85	1.81^a^	1.61
Chimpanzee	1.33^b^	1.71	0.78

a Obtained by mapping chimpanzee tracts to orthologous positions in the human genome.

b Obtained by mapping human tracts to orthologous positions in the chimpanzee genome.

At fine scales, the human and chimpanzee tracts show a modest, but significant, degree of overlap (Figure 4): 605 (6.4%) of the human tracts directly overlap a chimpanzee tract, compared with an average of 86 for the control regions (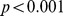). Shared recombination hotspots account for only a small minority (<1%) of the overlapping tracts. However, the correlation in tract locations between species is much stronger at broader scales. For example, if the fractions of nucleotides in gBGC tracts (“gBGC density”) are compared in orthologous genomic blocks of various sizes, the human/chimpanzee Pearson's correlation increases from 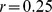 for 10 kb blocks to 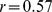 for 100 kb blocks, and to 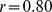 for 1 Mb blocks (Figure S7). These observations mirror those for human and chimpanzee recombination rates, which correlate well at scales of 1 Mb or larger but much more poorly at finer scales [23]–[25].

To gain further insight into the conservation of the gBGC tracts, we mapped the human gBGC tracts to orthologous locations in the chimpanzee genome, and the chimpanzee tracts to orthologous locations in the human genome. We then compared the recombination rates in these “ortho-tracts” with those in control regions, as with the tracts directly predicted for each species. Unlike recombination hotspots [25], the predicted gBGC tracts do show significantly elevated recombination rates at orthologous positions in the other species (Table 3). However, these recombination rates are not nearly as elevated as those for the directly predicted tracts. An analysis of the correlation between gBGC tract densities and recombination rates within and between species yielded similar results. Human gBGC tract densities are significantly correlated with human recombination rates, and this correlation increases with block size. A similar pattern is present in chimpanzee. When these correlations are examined across species (e.g., human gBGC densities vs. chimpanzee recombination rates), they are weaker but still significant (Figure S8). Differences in recombination rates between species are modestly predictive of differences in gBGC densities (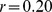 at 1 Mb; Figure S9). In general, we find much stronger correlations of gBGC- and recombination-associated features within species than between species, but these features nevertheless exhibit residual correlations between species, probably because they reflect average recombination rates over millions of years (see Discussion).

In both human and chimpanzee, the predicted tracts show a weak positive correlation with GC-content on a megabase scale. This correlation is somewhat stronger for human (Pearson's correlation for 1 Mb blocks: 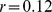) than for chimpanzee (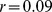), mirroring observations of a stronger correlation of recombination rate with GC-content in human than in chimpanzee [25].

### Genomic Annotations

To shed light on the functional implications of gBGC, we examined the degree of overlap of the predicted human gBGC tracts with various sets of genomic annotations (listed in Methods). In comparison with the control regions, we found that the human gBGC tracts were significantly depleted for overlap with known protein-coding exons, core promoters (1 kb upstream of annotated transcription start sites), miscellaneous RNAs, LINEs and SINEs, while they were significantly enriched for overlap with introns, lincRNAs, and a collection of ChIP-seq-supported transcription factor binding sites (Figure S10). However, all of these enrichments and depletions were modest in magnitude, with fold-changes of about 0.8–1.3. Overall, the gBGC tracts appear to be fairly representative of sequences of the same GC content. It is possible that the depletion for gBGC tracts in protein-coding exons and promoters could result in part from strong purifying selection counteracting GC-biased fixation.

### GC-Bias in Derived Alleles

To distinguish between fixation- and mutation-related biases, we compared the derived allele frequencies at polymorphic W→S and S→W sites in the predicted tracts and control regions. To control for the possibility of an ascertainment bias from polymorphic sites at which the derived allele is present in the human reference genome, we performed this analysis twice: once with the original gBGC tracts, and once with predictions based on alignments in which polymorphic sites in the human genome had been masked with ‘N’s.

**Figure 5:**
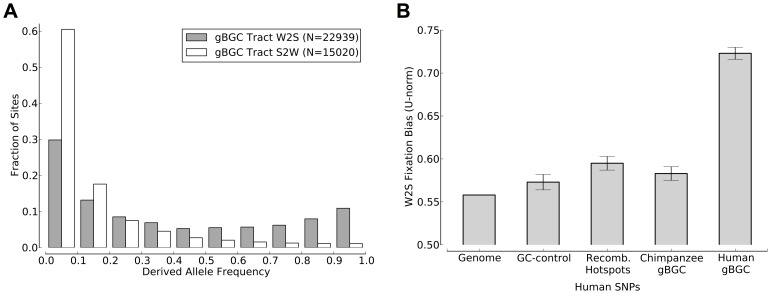
Human polymorphism data indicates an ongoing preference for the fixation of G and C alleles in the predicted gBGC tracts. (**A**) W→S changes in gBGC tracts have significantly higher derived allele frequencies than S→W changes in tracts. This plot is based on data for the YRI population from the 1000 Genomes Project [33]. Results for other populations were similar (data not shown). (**B**) The 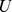-norm, a measure of the degree of W→S bias in polymorphism data [17], is significantly higher in gBGC tracts than in the entire genome or in GC-matched control regions (see Methods). Recombination hotspots also show somewhat elevated values but much less elevated than the predicted tracts. The 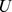-norm for human polymorphisms in “ortho-tracts” mapped from the chimpanzee genome is slightly elevated but significantly lower than that for human gBGC tracts. This is consistent with the lower human recombination rate in chimpanzee tracts compared to human tracts (Table 3). A similar species-specific skew in derived allele frequencies is seen in chimpanzee gBGC tracts (Figure S12). The error bars indicate 95% confidence intervals.

Based on pilot data from the 1000 Genomes Project [33] (YRI population), the predicted gBGC tracts displayed significantly elevated derived allele frequencies at sites of inferred W→S mutations compared with sites of inferred S→W mutations (W→S DAF skew of 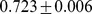; Figure 5A). This skew in DAFs was significantly greater than that observed genome-wide (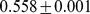) or in recombination hotspots (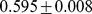; Figure 5B), and it was larger than observed in any of the 1000 control region replicates (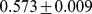). The tracts are also far more biased than any of the regions considered by Katzman et al. [17], which were identified using sliding windows of fixed size and likely contained a mixture of gBGC tracts and non-tracts. Results were similar for the CEU (W→S DAF skew of 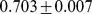) and CHB-JPT populations (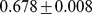). These results held for the tracts based on the polymorphism-masked alignments, although the magnitude of the skew was somewhat reduced in this case (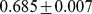 for YRI; Figure S11). Together, these results strongly indicate an on-going preference for the fixation of G and C alleles in the predicted gBGC tracts.

There is much less polymorphism data available for chimpanzees than for humans, but data for 10 individual chimpanzees from the PanMap project [25] indicates a similar ongoing fixation bias within the predicted chimpanzee tracts (Figure S12). As in human, the W→S DAF skew in the predicted chimpanzee tracts is significantly stronger than that observed in recombination hotspots. We also compared the W→S DAF skews of the tracts predicted for each genome and the “ortho-tracts” mapped from the other genome. As with recombination rates, we found that, in both species, the predicted tracts have significantly greater W→S DAF skews than the ortho-tracts (Figure 5B and Figure S12B). These findings are consistent with gBGC currently acting on a subset of our predicted tracts in association with transient, species-specific recombination hotspots.

### Fixation of Deleterious Alleles

Theoretical modeling has shown that gBGC, in principle, can overcome negative selection and result in the fixation of weakly deleterious alleles [3], [8], [10]. However, there is currently little direct empirical evidence of a contribution of gBGC to fixed or segregating deleterious alleles [11]. Our genome-wide tract predictions enabled us to investigate the link between gBGC and deleterious alleles by testing for enrichments for disease-associated genomic regions in gBGC tracts.

We examined the relationship between the gBGC tracts and four sets of putatively disease-associated genomic regions: 10,711 polymorphic sites from dbSNP annotated as “pathogenic” or “probable pathogenic” [34]; 43,952 polymorphic sites from the Human Gene Mutation Database (HGMD) [35] (see also [11]); 11,444 genomic regions from the Genetic Association Database (GAD) [36]; and 6,435,165 polymorphic sites with evidence of functional importance (classes 1–5) in RegulomeDB [37]. For the dbSNP pathogenic and HGMD comparisons, we considered sets of control regions that overlapped the same number of exonic SNPs as the gBGC tracts. This control is designed to avoid misleading findings of significance that simply reflect the GC content, exon coverage, and/or rates of polymorphism in the gBGC tracts, since these disease-associated region sets are mostly found in coding regions. Similarly, we used control regions matched to SNPs considered by RegulomeDB, since it only includes non-coding SNPs (Methods).

We found that the gBGC tracts overlapped significantly more putatively disease-related SNPs from the dbSNP, HGMD, and RegulomeDB collections, and significantly more of the GAD regions, than did the matched control regions (Table 4; 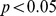 for each). In the cases of the two collections of disease-associated SNPs (dbSNP and HGMD), the enrichment within the predicted gBGC tracts was particularly striking (fold-enrichments of 2.4 and 1.9, respectively), while in the other cases it was more modest but still significant. These results suggest that gBGC may contribute in important ways to elevated allele frequencies, and perhaps, to the eventual fixation of deleterious mutations.

**Table 4:** Enrichment for disease-associated regions.

Disease-associated Region Set	gBGC Tract Overlap	Avg. Control Overlap	*p*-value
dbSNP Pathogenic	113	46.3	0.005
HGMD	346	178.2	0.031
RegulomeDB (classes 1–5)	26474	20768.4	<0.001
GAD	485	419.7	<0.001

### Overlap with Fast-Evolving Sequences

**Figure 6:**
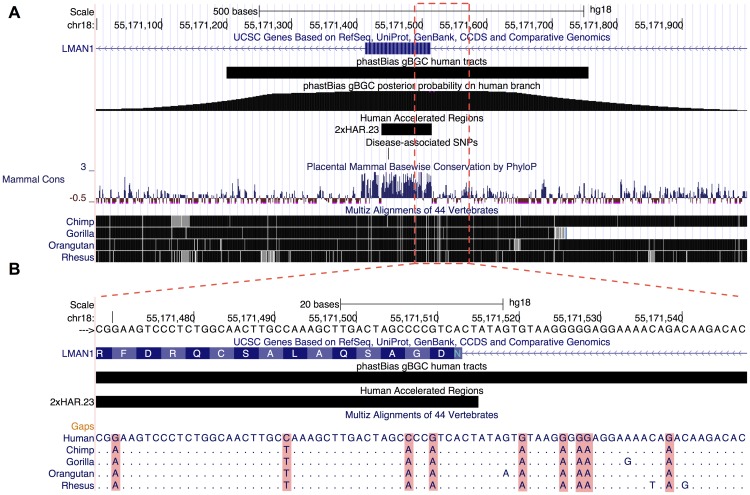
Illustration of genome browser track. (**A**) UCSC Genome Browser screen shot focused on the LMAN1 gene (hg18.chr18:55,148,088–55,177,461). This region contains a predicted gBGC tract (black bar, second track from top); the “wiggle” track below shows the posterior probability of gBGC at each site computed by phastBias. The gBGC tract overlaps an exon of the gene (blue bar at top; adjacent chevrons indicate introns), a human accelerated region (2×HAR.23; short black bar), and a known missense variant from dbSNP (rs146465318; black tick mark). The phyloP-based conservation track (“Mammal Cons”) shows that phastBias can predict tracts that span both conserved and nonconserved regions. The phastBias track is available at http://genome-mirror.bscb.cornell.edu (hg18 assembly). Notably, this region has an elevated recombination rate (2.5 cM/Mb; not shown). (**B**) The multiple sequence alignment for a portion of the gBGC tract (hg18.chr18:55,171,469–55,171,548) illustrates the characteristic signature of gBGC. This interval has nine human-specific W→S substitutions over 80 nucleotides, four of which fall within the exon. Positions in other species that match the human sequence are indicated with a period.

Many fast-evolving regions of the human genome display an excess of W→S substitutions, leading to the suggestion that gBGC may play a role in their evolution [6], [7], [9], [13], [38], [39]. Supporting this hypothesis, our predicted gBGC tracts overlap 13 of the 202 (6.4%) HARs identified by Pollard et al. [38], more than observed for any of the 1000 GC-control region replicates (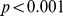). Notably, the HARs overlapped by gBGC tracts included HAR1, HAR2, and HAR3, the three fastest evolving sequences in this set. We also examined an expanded set of 721 HARs [40] and found that gBGC tracts overlapped 75 of them (10%; 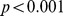; see example in Figure 6). Next, we compared the gBGC tracts with 10 protein-coding genes identified as showing signatures of positive selection on the human branch based on a likelihood ratio test [41]. One of these genes is overlapped by a gBGC tract, significantly more than expected based on exon-aware controls (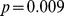). The overlapped gene, ADCYAP1, was also highlighted by another group [9] as showing strong evidence of an influence from gBGC. We repeated our analysis with 157 genes identified in another recent study as showing signatures of human-specific positive selection [42], and found that the gBGC tracts overlapped 11 (7%) of these genes, somewhat more than average for the exon-aware control replicates (7.4, 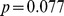). Considering our limitations in power (see Discussion), these results indicate the gBGC has contributed to a substantial fraction of fast-evolving sequences in the human genome.

### Genome Browser Track

Our predicted tracts for human and chimpanzee are available as a UCSC Genome Browser track at http://genome-mirror.bscb.cornell.edu (Figure 6). This track displays both our discrete predictions of gBGC tracts and a continuous-valued plot indicating the posterior probability that each position is influenced by gBGC. Using this track it is possible to browse the predicted tracts in their full genomic context, perform queries intersecting them with other browser tracks, and download them for further analysis. We expect this track to be particularly useful for other investigators who wish to exclude gBGC-influenced regions of the genome from other molecular evolutionary analyses, such as the identification of genes under positive selection. The tracts themselves will also be directly useful for studying the evolution of recombination rates and their relationship to substitution rates and patterns.

## Discussion

This paper describes an analysis of predicted gBGC tracts in the human and chimpanzee genomes, based on a new computational method called phastBias. PhastBias makes use of a hidden Markov model and statistical phylogenetic models that consider the influence of both natural selection and gBGC on substitution rates and patterns. Unlike previous methods for identifying signatures of gBGC, it does not depend on a sliding window or predefined annotations of protein-coding genes or conserved noncoding elements [9], [13], [15], [19], but instead can flexibly identify tracts of various sizes directly from genome-scale multiple alignments. The method appears to have good power for tracts of about 1 kilobase or longer, provided gBGC has acted with a reasonably high average intensity along the lineage of interest. Our predictions in the human and chimpanzee genomes cover about 0.3% of each genome and explain 1.2% of human/chimpanzee single nucleotide differences. Consistent with the hypothesis that they are caused by gBGC, the predicted tracts are correlated with recombination rates, tend to fall in subtelomeric regions, and exhibit an ongoing fixation bias for G and C alleles. In addition, they are enriched for disease-associated human polymorphisms, and they tend to overlap previously identified fast-evolving coding and non-coding regions, suggesting that gBGC has contributed significantly to both deleterious mutations and rapid sequence evolution. Overall, our analyses indicate that gBGC has been an important force in the evolution of human and chimpanzees since their divergence 4–6 million years ago.

Many attributes of the predicted gBGC tracts are consistent with the hypothesis that recombination is the driving force behind the observed patterns of biased substitution. Nevertheless, the tract locations are only partially correlated with recombination rates in human and chimpanzee. Moreover, while the tracts are enriched for recombination hotspots in both species, there are thousands of hotspots that do not overlap a gBGC tract, and the majority of tracts do not overlap a hotspot. These differences can be explained by several factors. First, the hotspots we have analyzed reflect recombination patterns in modern human populations, while the gBGC tracts reflect average patterns since the divergence of humans and chimpanzees. Many current hotspots presumably have not had sufficient time to produce a detectable signature of biased substitution, while many extinct hotspots contributed to gBGC for long periods of time in the past. Second, models of gBGC suggest that it can occur in conjunction with both crossover and noncrossover recombination events, but current recombination maps reflect crossover events only [3]. An imperfect correlation of these types of events, together with statistical noise in current estimates of crossover rates, likely accounts for some of the absence of correlation between recombination rates and gBGC tracts. Third, biased substitution rates are influenced by many factors other than recombination, such as mutation rates, natural selection, and GC content [43]. For example, strong purifying selection at or near a hotspot could eliminate the signature of gBGC. Finally, limitations in power for both recombination events and gBGC tracts undoubtedly reduce the apparent correlation between these features.

The locations of the human and chimpanzee tracts are strongly correlated on megabase scales, but, like recombination rates, they differ significantly on fine scales, and few human and chimpanzee tracts directly overlap one another (Figure 4; Figure S7). Nevertheless, even at fine scales, the human and chimpanzee gBGC tracts agree better than recombination hotspots, which are essentially uncorrelated between the two species [25]. This observation probably stems from the fact that gBGC tracts reflect time-averaged recombination rates, and historical recombination rates were presumably better correlated than those in present-day humans and chimpanzees. In general, the predicted gBGC tracts provide a valuable window into historical recombination processes, but this window is “blurred” by time-averaging over millions of years. Nevertheless, together with other sources of information about historical recombination processes—such as new methods based on patterns of incomplete lineage sorting (K. Munch, T. Mailund, J.Y. Dutheil, and M.H. Schierup, submitted)—predictions of gBGC tracts may help to provide a more detailed picture of the evolution of recombination rates in hominoids.

The different time scales associated with crossover-based recombination maps and our predicted gBGC tracts are particularly well illustrated by the region of the chromosome 2 fusion in human (Figure S6). Consistent with its location near a centromere in the human genome, this region displays no elevation of crossover rates in human populations, while the orthologous regions of the chimpanzee genome show elevated crossover rates typical of telomeres. Accordingly, this region exhibits little W→S DAF skew in human, but a clear skew in chimpanzee. However, the density of predicted gBGC tracts in this region is elevated in both species, only slightly more so in chimpanzee than human, suggesting that this region was telomeric for most of the approximately 6 million years during which human-specific recombination-associated substitutions could have occurred. Thus, our observations indicate that the fusion event is fairly old relative to intraspecies coalescence times but young relative to the human/chimpanzee divergence time. They are qualitatively consistent with Dreszer et al.'s [15] estimate of 0.74 Mya (95% confidence interval: 0–2.81 Mya) for the date of the fusion event and inconsistent with the argument that this event contributed to the initial speciation of humans and chimpanzees [44].

Despite the overall similarity of the human and chimpanzee predictions, the coverage of the predicted tracts is about 25% lower in the chimpanzee genome. A possible cause of this difference is the greater concentration of recombination events in hotspots in humans [25]. This difference could lead to a stronger population-level signal for gBGC in humans, allowing for more predictions and longer predicted tract lengths. It has been proposed that the difference in the concentration of recombination events may derive from differences in the activity of the hotspot-specifying protein PRDM9, which shows substantially greater allelic diversity in chimpanzees than in humans [25]. Consistent with this hypothesis, Auton et al. [25] found a much weaker signal for sequence motifs potentially involved in PRDM9 binding at chimpanzee hotspots than at human hotspots. In an attempt to shed light on the ancestral binding preferences of PRDM9, we applied motif discovery methods to the predicted gBGC tracts in the human and chimpanzee genomes. However, in both species this analysis turned up only a few motifs, none of which resembled the well-defined motifs reported for the human genome [25], [45]. This absence of strong motifs may occur because the ancestral recombination hotspots in both species are more like those in present-day chimpanzees than humans. Alternatively, it may simply reflect the difficulty of motif discovery given rapidly evolving PRDM9 binding preferences and the time-averaged nature of the gBGC tracts.

Given what is currently known about gBGC, it is impossible to obtain direct measurements of the completeness and accuracy of our predicted tracts. Our simulation experiments suggest that both sensitivity and specificity are reasonably good for tracts at least 1 kb in length with 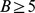, but we often miss shorter or less biased gBGC tracts (Figure 2), and the true distributions of tract lengths and 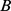 values are unknown (although average estimates of 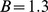 [46] and 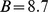 [8] have been reported for highly recombining regions). It is important to bear in mind that 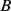 represents an average along an entire branch of the phylogeny. Many regions may have experienced quite strong gBGC but for short evolutionary intervals, resulting in small average values of 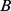 and poor detection power. Thus, while our genome-wide predictions improve on what is currently available, it seems plausible that they still represent the “tip of the iceberg”—a relatively small subset of all genomic regions significantly influenced by gBGC, perhaps unusual for their length or GC-disparity.

It is worthwhile to consider two other indirect sources of information about our power for gBGC tract prediction. First, Katzman et al. [17] found that about 20% of the 40 kb genomic intervals they examined show significant W→S DAF skew. If we conservatively assume one 1–2 kb tract per gBGC-influenced window, this observation would imply that at least 0.5–1.0% of the human genome has been influenced by gBGC on population genomic time scales, compared with the phastBias estimate (for 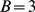) of 0.3%. Second, using a method optimized for the analysis of individual HARs, Kostka et al. [13] estimated that 24% of HARs experienced significant gBGC (19% exclusively and 5% in combination with positive selection), or 3.7 times as many as overlap our phastBias predictions (6.4%). Thus, these two imperfect indicators of power suggest that, with 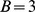, phastBias underpredicts gBGC tracts by a factor of at least about 2–4. The genomic coverage of our 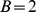 predictions may be closer to the truth (1.1%; Table 2), but these predictions appeared to be of poorer quality on inspection, apparently because the phylo-HMM states with and without gBGC were insufficiently distinct to control false positive rates.

While the likelihood ratio tests of Kostka et al. [13] appeared to have greater power for gBGC in HARs overall, phastBias sometimes achieves improved sensitivity by considering the entire genome (including flanking sequences) rather than just a designated collection of elements. Indeed, of the thirteen HARs that overlap one of our gBGC tracts, three were not identified by Kostka et al., apparently for this reason. These instances of improved sensitivity are especially noteworthy given that phastBias must address the more difficult problem of unconstrained genome-wide prediction, with the attendant potential for large numbers of false positives predictions.

In principle, gBGC can overcome purifying selection and help to drive deleterious alleles to high frequencies [3], [8], [10], but it has been difficult to find direct empirical evidence for a reduction in fitness (genetic load) caused by gBGC. Our predicted gBGC tracts are significantly enriched for disease-associated polymorphisms in current human populations, suggesting that gBGC has helped to drive at least some of these alleles to appreciable frequencies, and, indeed, may still be active in maintaining them. We attempted to establish an orthogonal link between gBGC and deleterious alleles by looking for evidence of purifying selection in chimpanzees and other species at the locations of W→S substitutions within the predicted human tracts (Text S1). The idea behind this analysis was that, if a substantial number of these mutations were driven to fixation by gBGC despite negative selection against them, one would expect an excess of evolutionary conservation, a deficiency of polymorphisms, and/or a skew toward low-frequency derived alleles at orthologous locations in other species, relative to an appropriate control. However, this analysis yielded inconclusive results: the human tracts are significantly enriched for overlap with evolutionarily conserved elements at locations of W→S substitutions (Figure S13), but evolutionary conservation scores and chimpanzee polymorphisms do not display the expected patterns (Figures S14, S15, and S16). It seems likely that the signal for excess conservation in the gBGC tracts is simply too weak to detect by these methods, owing to the sparseness of functional sites within the tracts and the difficulty of establishing appropriate control regions. Nevertheless, it may be possible in future work to develop refined comparative genomic methods for measuring the genetic load associated with gBGC.

## Methods

### Probabilistic Model

Our phylogenetic hidden Markov model has four states: one that assumes both evolutionary conservation and gBGC (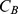), a second with gBGC but no conservation (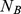), a third with conservation but no gBGC (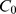), and a fourth with neither conservation nor gBGC (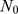) (Figure 1). To avoid over-parameterization, we make the following simplifying assumptions. First, we model gBGC only on the lineage leading to a pre-defined “target” genome (human or chimpanzee), because gBGC is expected to be a transient phenomenon, typically affecting a single lineage in any genomic position of interest. gBGC tracts are allowed to occur on other lineages, but these tracts are expected to have a negligible influence on inferences in the target genome and are not directly modeled. Second, negative selection, in contrast to gBGC, is assumed to apply uniformly across all branches of the phylogeny. Third, positive selection is ignored. We omit positive selection and lineage-specific negative selection from the model because they are expected to be fairly rare, to leave a relatively weak signal in the data at human-chimpanzee evolutionary distances [47], and to primarily operate at a somewhat different genomic scale from gBGC (e.g., at the level of individual binding sites or clusters of amino acids, rather than genomic tracts of hundreds or thousands of bases). We expect our modeling framework to be robust to occasional sequences under positive or lineage-specific selection, because the primary signal for tract prediction is a W→S substitution bias, and selection generally will not produce such a bias consistently across many bases. Finally, we assume that the strength of gBGC and the strength of negative selection in the target genome are constant across the genome. A similar homogeneity assumption is employed in phastCons and appears to have a minimal impact on power and accuracy for element identification [28].

With these assumptions, the phylogenetic models for the four states are defined as follows (with further mathematical details given in Text S1).

1. Neutral/No gBGC (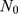): Neutral evolution is described by an HKY substitution model [48], with free parameters for the transition/transversion ratio (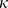) and stationary nucleotide frequencies (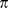). We assume the accepted tree topology for the species under consideration: (((human, chimpanzee), orangutan), rhesus macaque). The branch length proportions were obtained from the Conservation tracks in the UCSC Genome Browser (assembly hg18) [49]. (They were originally estimated from fourfold degenerate sites in protein coding genes under a strand-symmetric general reversible model.) These branches were scaled locally to accommodate regional variation in mutation rate (see below).
2. Neutral/gBGC (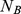): This model is identical to the neutral model except that it assumes gBGC influences substitution rates and patterns on the lineage leading to the target species (human or chimpanzee) according to the model of Kostka et al. [13]. The strength of gBGC is described by the GC-disparity parameter 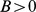, which increases the rate of W→S substitutions and decreases the rate of S→W substitutions.
3. Conserved/No gBGC (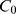): Evolutionary conservation is modeled, as in phastCons, by multiplying the branch lengths of the neutral model by a factor 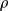 (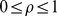). In all other respects, this model is identical to the neutral model.
4. Conserved/gBGC (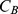): This model is identical to model 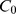 except that it assumes gBGC acts with strength 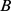 on the lineage leading to the target species.

The state-transition probabilities are defined by four parameters, denoted 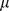, 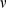, 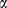, and 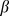 (Figure 1, Table 1). The parameters 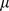 and 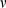 are inherited from phastCons [28] and describe the conditional probabilities of transitioning from a conserved state to a neutral state, and from a neutral state to a conserved state, respectively. The parameters 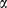 and 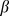 are analogous, defining the conditional probabilities of transitioning out of, and into, a gBGC tract, respectively. The sixteen possible state transition probabilities are obtained by multiplying the appropriate pairs of conditional probabilities and enforcing the standard normalization constraints (Figure 1). This “cross-product” construction corresponds to a prior assumption of independence for the two types of transitions (conservation 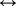 no conservation and gBGC 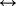 no gBGC).

Given a multiple sequence alignment, standard algorithms for statistical phylogenetics and hidden Markov models can be used to calculate the likelihood of the data under this model, to predict the most likely state path (Viterbi), or to calculate the marginal posterior probability of each state at each alignment column (reviewed in [27]).

### Parameter Estimation

In principle, the nine free parameters in our model (Table 1) could all be estimated directly from the data by maximum likelihood, using an expectation maximization or numerical optimization algorithm. In practice, however, parameter estimation is difficult because there are no validated gBGC tracts to use for supervised training of the model, and the signal in the data is not sufficiently strong to support a fully unsupervised estimation procedure. Instead, we partition the parameters into three groups: those for the neutral substitution process, those for the model of conserved elements, and those specific to the gBGC tracts. The first two groups of parameters are pre-estimated from the data without consideration of gBGC, by what can be considered an empirical Bayes approach. The parameters in the third group are then estimated by a combination of methods.

Specifically, the free parameters for the neutral substitution process (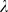, 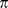, and 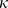) are estimated per alignment block (see below) using phyloFit [26], after conditioning on the tree topology and branch-length proportions (as described above). This strategy assumes that conserved elements and gBGC tracts are sparse and have at most a minor effect on average substitution rates for large genomic blocks. The three additional parameters that describe conserved elements (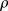, 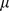, and 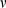) are inherited directly from phastCons and therefore were simply set to the values used for the Conservation tracks in the UCSC Genome Browser. The remaining parameters include the GC-disparity 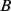 and the gBGC transition probabilities 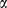 and 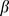. As discussed in the Results section, we found that 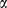
—which can be interpreted as an inverse prior expected length for gBGC tracts—has only a weak influence on our predictions (within a reasonable range) and decided to simply fix it at 1/1000, corresponding to a prior expectation of 1 kb tracts. We treated 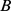 as a “tuning” parameter and considered various possible values in a plausible range. The final parameter, 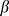, was estimated from the data (separately for each alignment block) by expectation maximization, conditional on fixed values of all other parameters.

### Tract Prediction

To predict gBGC tracts based on our model, we computed marginal posterior probabilities for the four model states at each genomic position using the forward/backward algorithm. We then computed the marginal posterior probability of gBGC by summing the probabilities for states 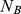 and 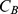, and we predicted tracts by applying a threshold of 0.5 to this probability (i.e., the predicted tracts are maximal segments in which every position has a posterior probability of at least 50% of gBGC). We settled on this strategy after discovering that the more conventional Viterbi algorithm performed poorly in this setting, evidently due to uncertainty about the endpoints of tracts. This uncertainty causes the probability mass for a putative gBGC tract to be distributed across many possible HMM state paths, and as a result, the Viterbi algorithm often fails to predict a tract even when the posterior probability of gBGC is close to one. A potential drawback of our thresholding strategy is that fluctuating posterior probabilities could lead to highly fragmented tract predictions. However, we found that the posterior probability function was quite smooth in practice (probably owing to small values of the state transition probabilities) and fragmentation was not a problem. For example, at 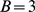, only about 2% of the predicted human tracts fall within 50 base pairs of another tract. Nonetheless, when analyzing the genomic distribution of gBGC tracts relative to one another and to telomeres, we merged adjacent tracts (within 1 kb) in order to reduce any bias introduced by over fragmentation (Text S1).

### Genome-Wide Alignments and Preprocessing

Our analyses of both simulated and real data were based on genome-wide alignments obtained from the UCSC Genome Browser (http://genome.ucsc.edu) [49]. We began with the 44-way vertebrate alignments produced with multiz [50] (hg18 assembly) and extracted the rows corresponding to the human, chimpanzee, orangutan, and rhesus macaque genomes, discarding alignment columns containing only gaps in these sequences. We also discarded columns in which the human genome contained a gap. Human-referenced alignments were used for both the human and chimpanzee gBGC tract predictions, as chimpanzee-based multiple alignments are not available. For convenience in processing, the resulting four-way alignments were partitioned into blocks of approximately 10 megabases (Mb) in length. The boundaries between blocks were required to occur in regions uninformative about gBGC (due to greater than 1 kb with lack of alignment with the other species). We experimented with several alternative block sizes, ranging from 1–30 Mb, and found that the predictions were fairly robust to the choice of block size (Table S2).

### Simulation Study

We simulated human sequences with gBGC tracts for each 10 Mb block in the real genome-wide alignments as follows. First, we identified positions at which any sequence contained a CpG dinucleotide, because substitution rates are likely to be substantially elevated at such sites. Next, we used phastCons to identify conserved elements in the four species. We then fitted a phylogenetic model to the alignment columns in each of four categories (neutral/non-CpG, conserved/non-CpG, neutral/CpG, conserved/CpG) by estimating 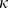, 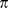, and 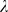 for the most data-rich category (neutral/non-CpG), then estimating a separate 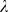 for the CpG category (using phyloFit) and applying a branch-length scale-factor of 0.31 to the conserved categories. Next, we defined an alternative “gBGC” instance of each of the four estimated models by modifying the substitution rate matrix for the human branch according to our model of gBGC [13] and a given choice of 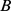 (here denoted 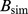). In this way, we obtained eight phylogenetic models, representing all combinations of conservation/no conservation, CpG/no CpG, and gBGC/no gBGC.

We generated synthetic human sequences by assigning one of these eight models to each alignment column, as follows. The conservation and CpG status of each column was maintained as originally annotated, so that the synthetic alignments would resemble the original ones as much as possible. The gBGC status was set to “no gBGC” for most columns, but set to “gBGC” for tracts of fixed size at randomly selected locations, at an average gBGC coverage of 0.1%. We then simulated a new human base for each alignment column conditional on the assigned phylogenetic model and the observed chimpanzee, orangutan, and rhesus macaque bases. This was accomplished using the ‘postprob.msa’ function in RPHAST, which computes the marginal distribution over bases at any node in the phylogeny conditional on a given phylogenetic model and collection of observed bases, using the sum-product algorithm. This function computes the desired distribution for the human base if the human sequence is masked and treated as missing data in the input. A particular base was selected by sampling from this marginal distribution.

We performed this simulation procedure for combinations of 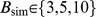 and fixed tract lengths of 200, 400, 800, 1600, 3200, and 6400. For each set of simulated alignments, we predicted gBGC tracts as described in the previous section, assuming several different values for the tuning parameter 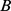. For each data set and value of 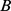, we calculated the true positive rate (number of correctly predicted gBGC bases/total number of gBGC bases), false positive rate (number of incorrectly predicted gBGC bases/total number of non-gBGC bases), and positive predictive value (number of correctly predicted gBGC bases/number of predicted gBGC bases).

### Genomic Annotations

We compared the predicted gBGC tracts with exon and intron definitions from Gencode version 3c and Ensembl genes [51], and with annotations of lincRNAs, miRNAs, miscRNAs, small non-coding RNAs, NMD transcripts, and pseudogenes from Gencode version 14 [52]. We also compared them with LINE and SINE elements from the rmskRM327 table in the UCSC Table Browser [53], and with a set of high-confidence predictions of transcription factor binding sites based on ChIP-seq data from ENCODE [54]. In addition, we compared the tracts with genome-wide recombination rate estimates from the 1000 Genomes Project [33], recombination hotspots from the October 2006 release of HapMap [32], and chimpanzee recombination rate estimates from the PanMap project [25].

Disease-associated SNPs were obtained from several sources. SNPs annotated with “pathogenic” or “probable pathogenic” clinical significance were downloaded on October, 25, 2011 from dbSNP [34]. The HGMD dSNPs were obtained from the Supplementary Material of reference [11]. Regions of the human genome with positive genetic associations with disease were taken from the Genetic Association Database [36] on February 2, 2012. The level of evidence for the function of non-coding SNPs was downloaded from the RegulomeDB [37] web site on December 12, 2012. All data not in reference to the GRCh36/hg18 assembly were mapped to hg18 using the ‘liftOver’ tool from the UCSC Genome Browser.

### Control Regions

To evaluate the statistical significance of various properties of interest, we compared the predicted gBGC tracts with sets of control regions matched to them in number, length distribution, and chromosome assignment. We also ensured that the control regions were matched to the gBGC tracts by GC content (by stratifying predictions and controls into 100 bins), which is known to correlate strongly with several relevant genomic features. We obtained a null distribution for each statistic of interest (such as the number of tracts overlapping exons, or the number human tracts overlapping orthologous chimpanzee tracts), by computing a value of the statistic for each of 1000 randomly sampled replicates of the control regions. One-sided empirical p-values were computed as the fraction of sampled control sets for which the statistic was at least as extreme as observed in the predicted tracts. As noted in the text, we occasionally considered alternative sets of control regions designed to accommodate known biases in genomic regions of interest. For example, when evaluating the significance of overlap with disease-associated SNPs from HGMD and dbSNP, we used control regions matched to the predicted tracts in terms of their degree of exon overlap, since these sets consist mostly of coding SNPs. Similarly, for RegulomeDB, which is focused on non-coding SNPs, we used control regions that matched the overlap of the gBGC tracts with the set of SNPs considered by RegulomeDB.

### Analysis of Derived Allele Frequencies

Our analysis of human derived allele frequencies was based on genotype data and ancestral allele predictions from the low-coverage pilot data set from the 1000 Genomes Project released in July 2010 [33]. These comprise SNP calls for the 22 autosomes in three HapMap population panels: YRI (59 individuals), CEU (60 individuals), and CHB-JPT (60 individuals). The chimpanzee derived allele frequency analysis was based on genotype data for 10 individuals downloaded from the PanMap project [25]. SNP locations were mapped to the human genome, and the 1000 Genomes predicted human chimpanzee ancestral allele was used to identify the derived allele. Sites with a low quality genotype call (GQ quality score less than 5), more than two alleles, or no predicted ancestral allele were not considered. We computed the W→S DAF skew of all human and chimp gBGC tract SNPs as normalized 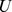 values from a Mann-Whitney 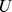 test on the derived allele frequencies of W→S and S→W SNPs, as previously described [17]. A W→S DAF skew of 0.5 indicates no bias, and values greater than 0.5 indicate that W→S mutations are favored.

## Supporting Information

Figure S1False positive rates from simulations. Each point in the plot represents the false positive rate obtained by analyzing a set of simulations in which all tracts have the same strength and length. The solid lines show the total false positive rate, calculated as the total fraction of bases outside of gBGC tracts that were assigned to gBGC tracts by phastBias using 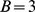. The dashed lines show the false positive rates only counting predicted tracts which do not overlap simulated tracts. Most false positives come from uncertainty in the tract boundaries, especially for short tracts.(PDF)

Figure S2Additional simulation results. Power and accuracy for gBGC tract prediction as a function of (**A**) gBGC strength (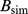), (**B**) the tuning parameter 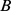, (**C**) mean tract coverage, and (**D**) mean tract length. Solid lines represent basewise true positive rate (TPR) and dotted lines represent positive predicted value (PPV). In each plot, tracts were simulated with 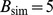, a geometric length distribution with a mean of 1 kb, and mean coverage of 1%, unless otherwise specified by the x-axis. The phylo-HMM was run with the same parameter settings used for the genome-wide predictions, including 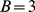, except in (**B**) (where 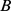 is varied).(PDF)

Figure S3W→S bias distribution for human substitutions in gBGC tracts for 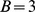. Histogram of W→S bias, which is computed for each tract as the fraction of all W→S and S→W substitutions along the human lineage which are W→S. Human-chimpanzee substitutions were polarized by assuming the allele observed in orangutan (ponAbe2) was ancestral.(PDF)

Figure S4 Distance to telomere and recombination rate correlate with gBGC-tract proximity. This figure shows box plots of the distribution of the log distance to the nearest gBGC tract, stratified by log distance to nearest telomere (first column) and recombination rate (second column) for both human (first row) and chimp (second row). For both species we observe that gBGC tracts are closer together towards the end of chromosomes (panels **A** and **C**), and that they are further apart in areas of low recombination rate (panels **B** and **D**). These empirical observations agree with the results of our linear modeling analysis (Text S1).
(PDF)

Figure S5 gBGC tracts are clustered and closer to telomeres than expected by chance. This figure shows qq-plots contrasting quantiles observed in gBGC tracts (x-axis) with medians of quantiles observed across GC-matched control sets (points, y-axis). The gray regions correspond to the data range observed across control regions (with the 1% highest and 1% lowest values removed). The vertical blue dashed line denotes the median for the gBGC tracts. Panels **A** and **B** show these plots for distance to nearest gBGC tract and the distance to nearest telomere in human; **C** and **D** show the corresponding plots for chimpanzee.
(PDF)

Figure S6Signatures of recombination around the fusion site on human chromosome 2. Shown are predicted gBGC tract densities per megabase (top), crossover rates [25], [33] (middle), and DAF skews (bottom; see Methods) for a 20 Mb region centered on the fusion site on human chromosome 2 (gray vertical line). Separate lines represent data from the human genome (black) and the orthologous regions of chromosomes 2a and 2b in the chimpanzee genome (red). All measures are standardized by subtracting the chromosome-wide mean and dividing by the standard deviation. The raw data were smoothed using a Gaussian filter with 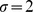. See the Discussion for interpretation of these differences between human and chimpanzee.(PDF)

Figure S7 Human and chimpanzee gBGC tracts are found in broadly similar locations, but exhibit fine-scale differences. The fraction of bases in gBGC tracts is correlated between human and orthologous chimpanzee regions (Figure 4). The strength of this correlation increases as larger blocks of the genome are considered (x-axis). The gray bars give the average and standard deviation of the correlations observed between the gBGC fraction in 1000 GC-matched human control regions and the orthologous chimpanzee regions.
(PDF)

Figure S8 Correlation of recombination rates and gBGC tract densities within and between species. Recombination rates and gBGC densities are significantly correlated within species, and this correlation is more pronounced at larger scales (dark blue and dark red bars). When gBGC tract densities and recombination rates are compared across species (human gBGC tract densities vs.chimpanzee recombination rates or chimpanzee gBGC tract densities vs.human recombination rates; light blue and light red bars, respectively) they show weaker but still significant correlations. This plot considers only blocks that have nonzero values for all four statistics of interest.
(PDF)

Figure S9Differences in gBGC tract density between human and chimpanzee are modestly correlated with differences in recombination rate. Bars show the Pearson correlation between the difference between 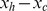 and 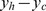, where 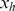 and 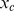 are the human and chimpanzee gBGC tract densities, respectively, and 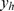 and 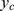 are the human and chimpanzee recombination rates, respectively. Average values were computed for windows of various sizes (x-axis). All correlations are significantly greater than zero (10 kb: *p* = 0.01; 100 kb *p* = 6.5e–05; 1 Mb: *p* = 6.7e–11; 5 Mb: *p* = 1.7e–07).(PDF)

Figure S10Genomic features significantly enriched or depeleted in gBGC tracts. For each genomic feature, we compared the number of overlaps observed with gBGC tracts with those observed in 1000 random GC-matched control regions. The gray bars give the minimum and maximum overlaps observed in the random sets. Shown are all features that are significantly (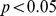) underrepresented (blue) or overrepresented (red) in the tracts. See the Methods for a full list of genomic features considered. Note that the tracts are more strongly enriched for recombination hotspots (not shown, 1.54×) and for high recombination rates (Table 3), both of which were considered separately.(PDF)

Figure S11Human polymorphism data indicate an ongoing preference for the fixation of G and C alleles in the predicted gBGC tracts. This figure shows the same plots as Figure 5, but is based on an analysis in which polymorphic sites were masked from the alignments. (**A**) W→S changes in gBGC tracts have significantly higher derived allele frequencies than S→W changes. This result was obtained on the YRI population from the 1000 Genomes Project, and patterns for other populations were similar (data not shown). (**B**) The 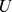-norm, a measure of the degree of W→S bias (see Methods), is significantly higher in gBGC tracts than in the entire genome or in GC-matched control regions. Recombination hotspots also show somewhat elevated values but much less elevated than the predicted tracts. The error bars indicate 95% confidence intervals.(PDF)

Figure S12Chimpanzee polymorphism data indicate an ongoing preference for the fixation of G and C alleles in the predicted chimpanzee gBGC tracts. This figure shows the same analysis as Figure 5, but is based on chimpanzee polymorphism data for 10 individuals (20 chromsomes per site) from the PanMap project. (**A**) W→S changes in chimpanzee gBGC tracts have significantly higher derived allele frequencies than S→W changes. (**B**) Echoing the bias patterns observed in human polymorphism, the 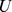-norm, a measure of the degree of W→S bias (see Methods), is significantly higher in chimpanzee gBGC tracts than in the entire genome and human gBGC tracts mapped to the chimp genome. Chimpanzee recombination hotspots also show somewhat elevated values but much less elevated than the predicted tracts. The error bars indicate 95% confidence intervals.(PDF)

Figure S13W→S sites within the predicted human tracts are enriched for phastCons elements compared to controls. Enrichments were calculated as the number of W→S substitutions within tracts falling in phastCons elements, divided by the number expected if these were distributed independently. The histograms show enrichment in our sets of 1000 GC- and exon-aware control tracts, and the arrow shows the value observed in the 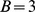 gBGC tracts.(PDF)

Figure S14 Conservation at sites of W→S substitutions within tracts. PhyloP scores were calculated at sites within the predicted human tracts at which W→S substitutions occurred on the human lineage. They were also calculated at sites of similar human-specific W→S substitution within the GC- and exon-matched control groups (1000 replicates). The scores were calculated for mammalian alignments from which the human and chimpanzee sequences had been removed. A higher phyloP score (x-axis) indicates greater evolutionary conservation. Although there are slight differences between the distributions for the tracts and the control groups, there is no clear excess of conservation at W→S sites in the tracts.
(PDF)

Figure S15 Number of chimpanzee polymorphisms in regions orthologous to the gBGC tracts, compared to controls. We observed significantly more chimpanzee polymorphisms in regions orthologous to the tracts than those orthologous to the control groups. This is the opposite of the observation that would be expected if the regions orthologous to the tracts were under purifying selection in the chimpanzee.
(PDF)

Figure S16Derived allele frequency spectrum of chimpanzee polymorphisms in regions orthologous to the tracts. The top plot shows the derived allele frequency spectrum (polarized using the orangutan allele) for chimpanzee polymorphisms in regions orthologous to the 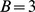 gBGC tracts, compared with the minimum and maximum from 1000 control groups. The bottom plot shows the fraction of samples from each control group with a higher frequency than observed in the real tracts. We observe no significant excess of low-frequency derived alleles in regions orthologous to the tracts.(PDF)

Table S1Relative coverage of human gBGC tracts for various values of *B*. Each value in the table represents the fraction of nucleotides in the human gBGC tract predictions for the value of 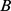 indicated for the row that also fall in the predictions for the value of 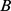 indicated for the column. The numbers on the main diagonal are one by definition. The numbers above the main diagonal indicate the coverage of smaller sets (higher 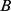) by larger sets (lower 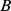), while the numbers below the main diagonal indicate the coverage of larger sets by smaller sets.(PDF)

Table S2 Relative coverage of tracts predicted using various block sizes. This table shows the robustness of tract predictions to the block size used in analysis. The tracts presented in the paper were computed in 10 Mb blocks (highlighted). As discussed in the Methods section, several free parameters were estimated separately for each block, including the rate into the gBGC state. In each row of this table is shown the fraction of bases predicted at a given block size which were also predicted using the block size indicated by the column header. Most pairs of block size choices have overlaps of greater than 80%, except for the case of 1 Mb blocks, where some overfitting appears to occur. Inspection of the predictions indicates that most differences between predictions are due to short tracts for which the posterior probability for gBGC is near the threshold of 50%.
(PDF)

Text S1 Text describing additional analyses in support of the manuscript.
(PDF)
