# Supplementary figures and images for: A Model-Based Analysis of GC-Biased Gene Conversion in the Human and Chimpanzee Genomes
Source: PLoS Genet. 2013 Aug 15;9(8):e1003684. doi: 10.1371/journal.pgen.1003684 (PMC3744432; doi:10.1371/journal.pgen.1003684)

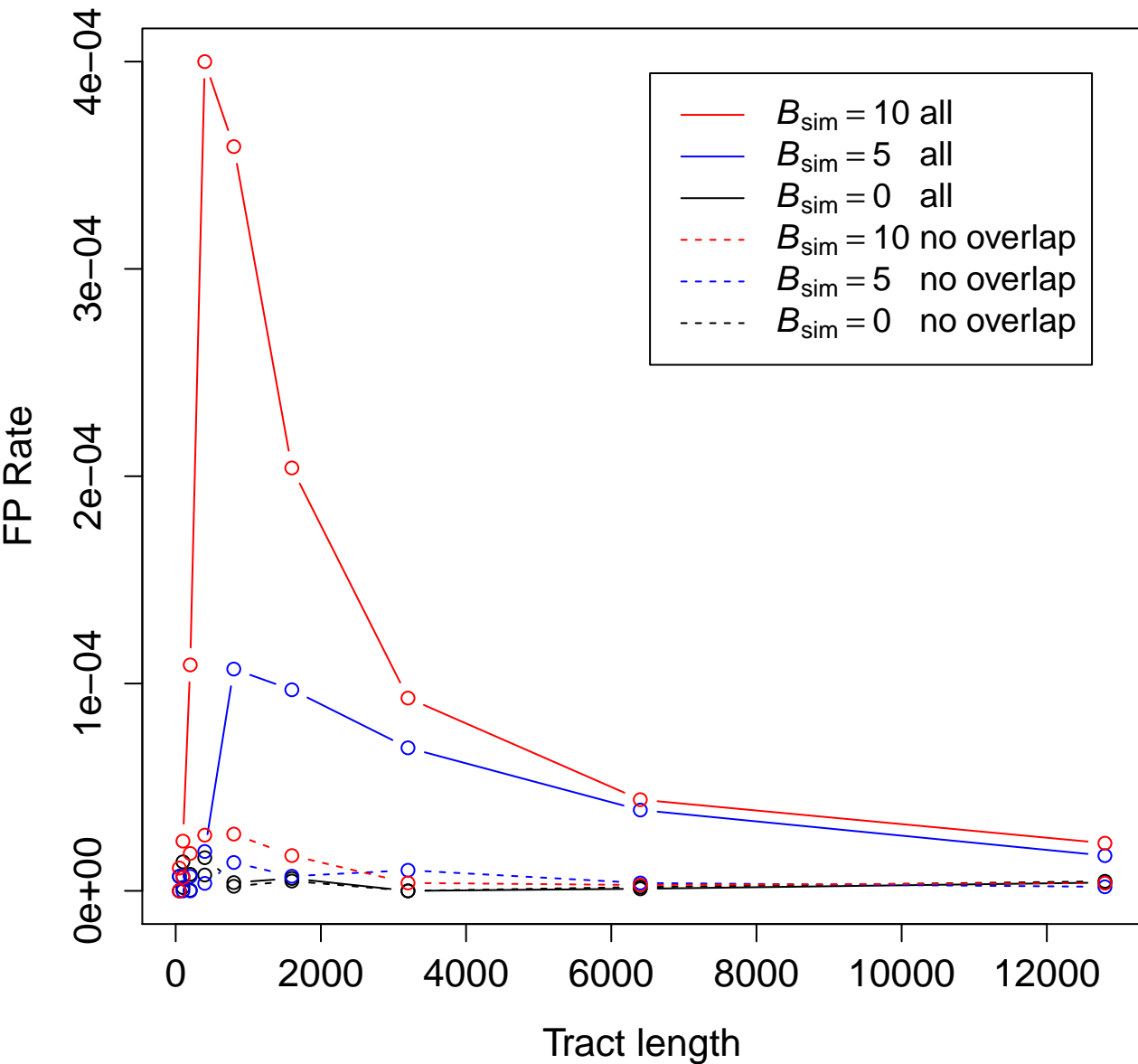

Supplement: Figure S1 — False positive rates from simulations. Each point in the plot represents the false positive rate obtained by analyzing a set of simulations in which all tracts have the same strength and length. The solid lines show the total false positive rate, calculated as the total fraction of bases outside of gBGC tracts that were assigned to gBGC tracts by phastBias using . The dashed lines show the false positive rates only counting predicted tracts which do not overlap simulated tracts. Most false positives come from uncertainty in the tract boundaries, especially for short tracts. (PDF) [file pgen.1003684.s001.pdf]

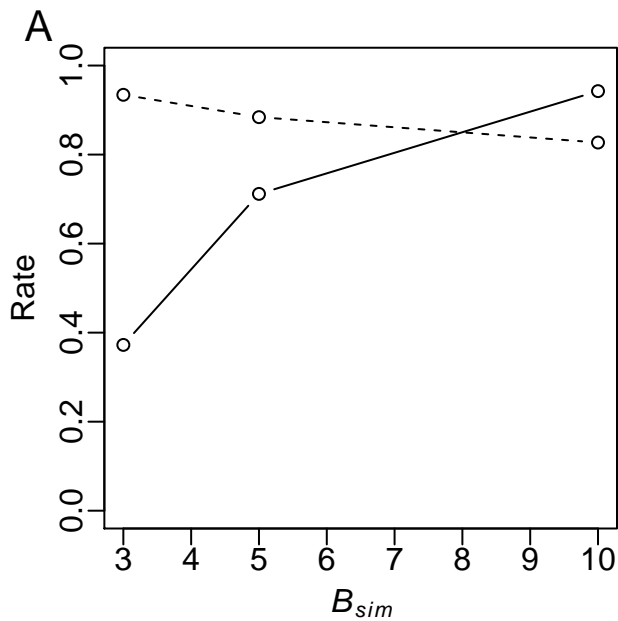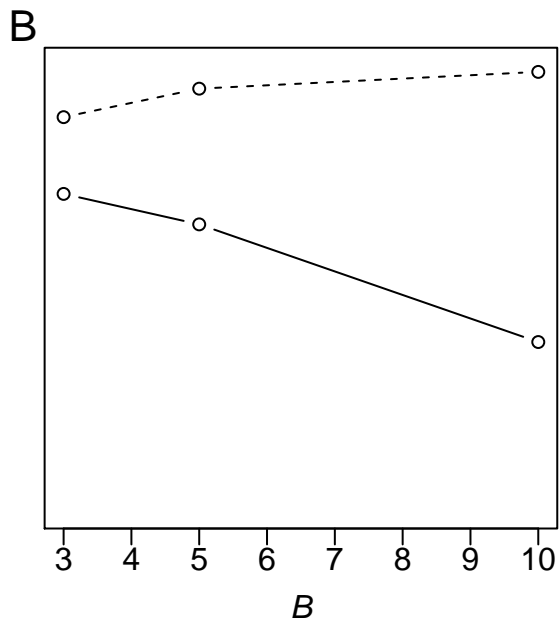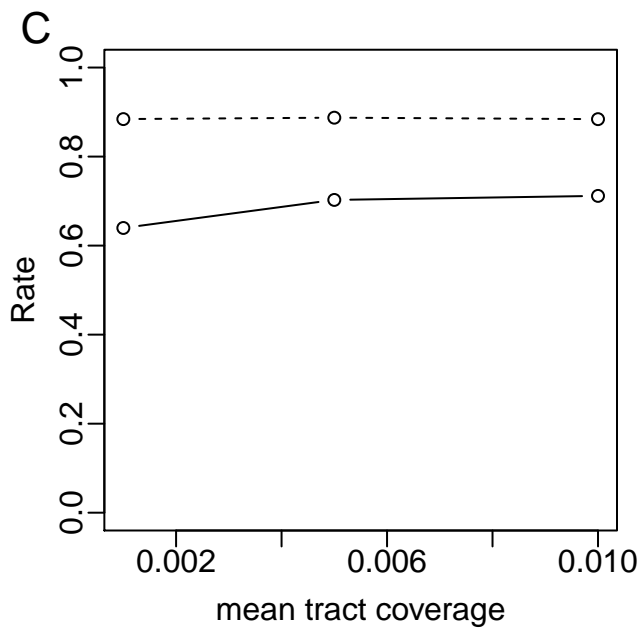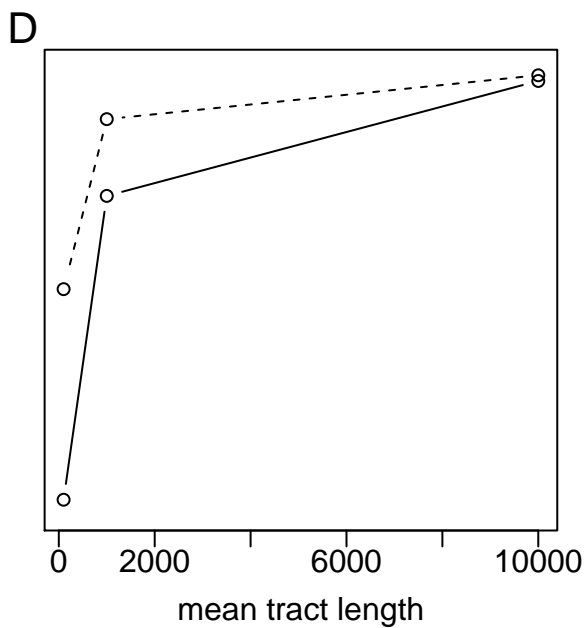

Supplement: Figure S2 — Additional simulation results. Power and accuracy for gBGC tract prediction as a function of (A) gBGC strength (), (B) the tuning parameter , (C) mean tract coverage, and (D) mean tract length. Solid lines represent basewise true positive rate (TPR) and dotted lines represent positive predicted value (PPV). In each plot, tracts were simulated with , a geometric length distribution with a mean of 1 kb, and mean coverage of 1%, unless otherwise specified by the x-axis. The phylo-HMM was run with the same parameter settings used for the genome-wide predictions, including , except in (B) (where is varied). (PDF) [file pgen.1003684.s002.pdf]

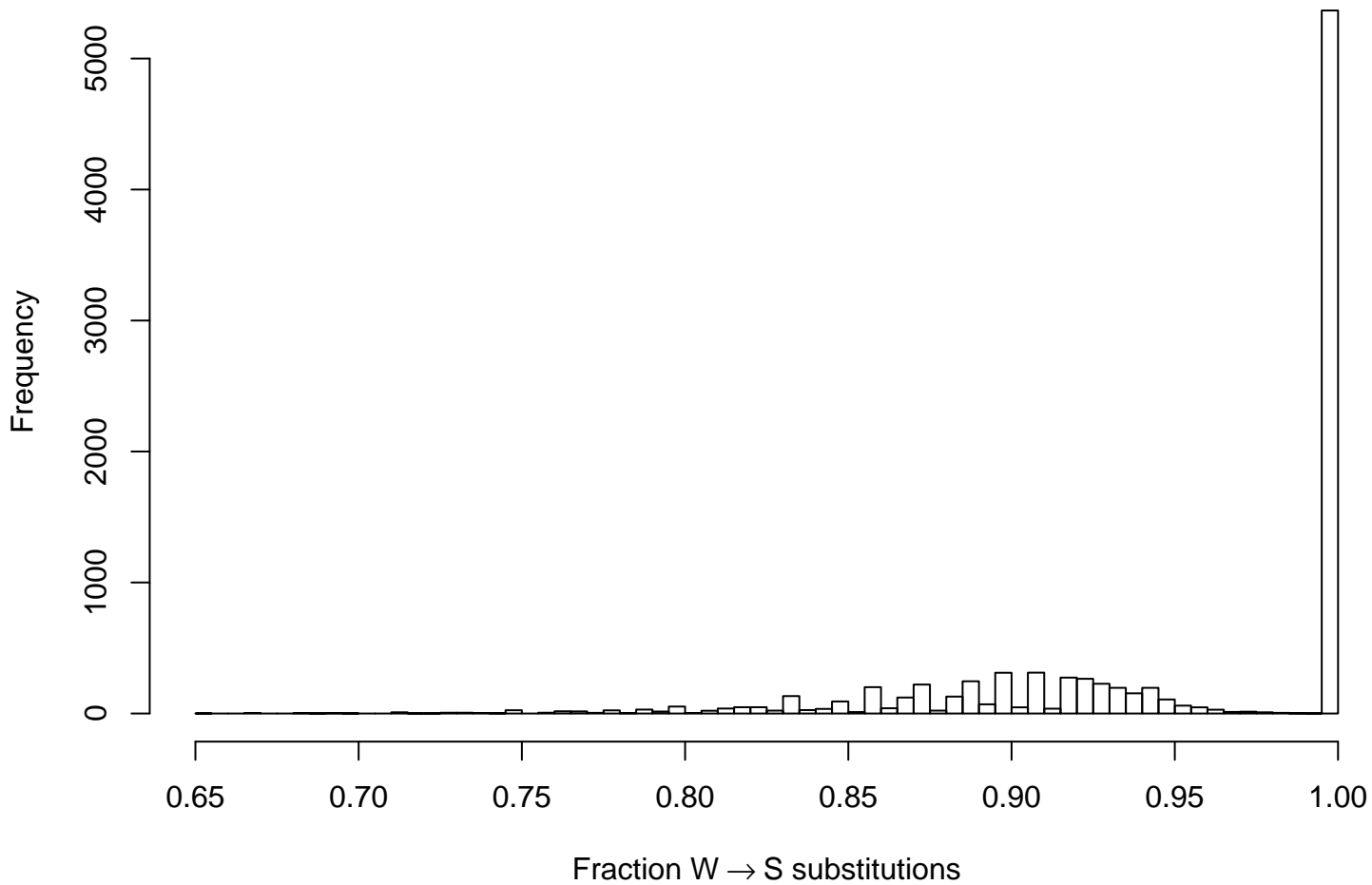

Supplement: Figure S3 — W→S bias distribution for human substitutions in gBGC tracts for . Histogram of W→S bias, which is computed for each tract as the fraction of all W→S and S→W substitutions along the human lineage which are W→S. Human-chimpanzee substitutions were polarized by assuming the allele observed in orangutan (ponAbe2) was ancestral. (PDF) [file pgen.1003684.s003.pdf]

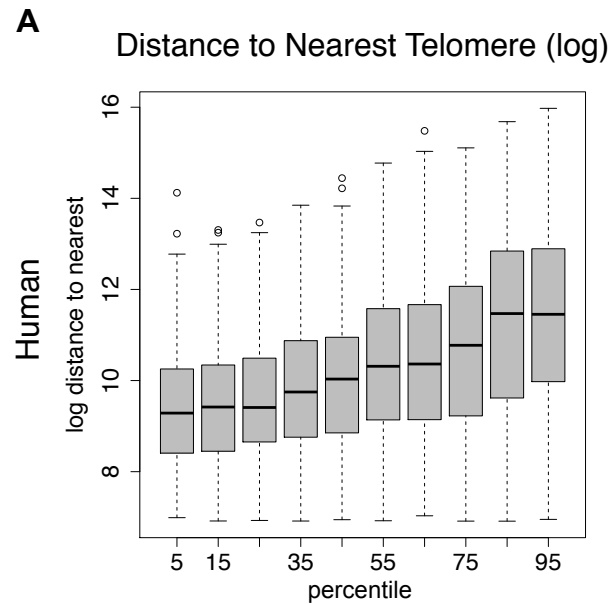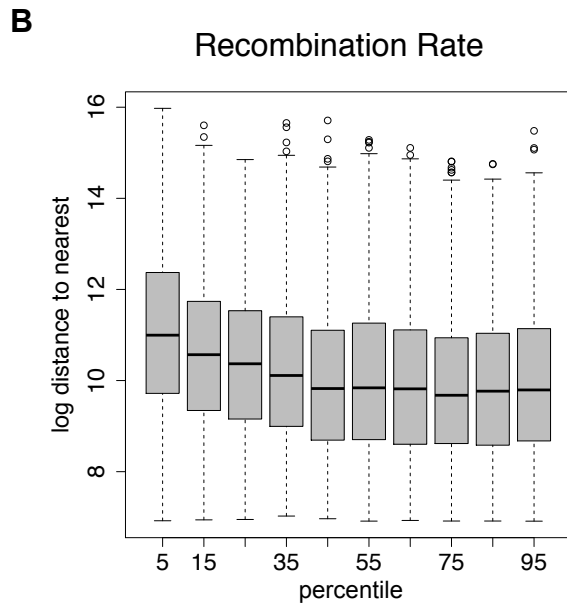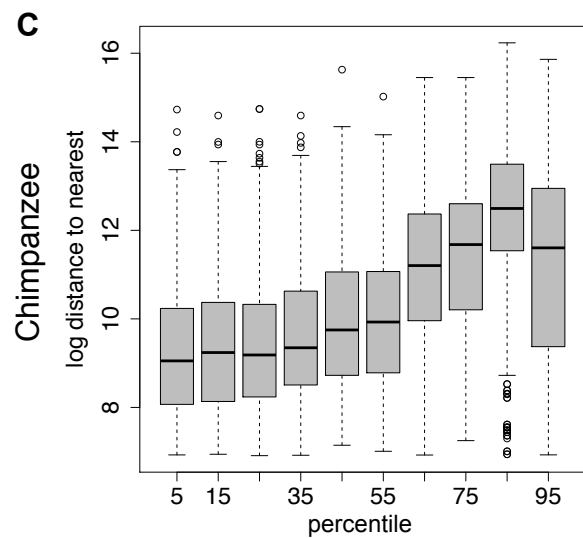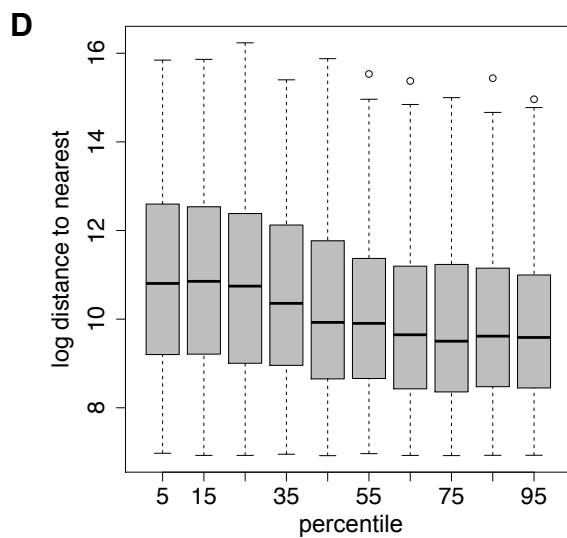

Supplement: Figure S4 — Distance to telomere and recombination rate correlate with gBGC-tract proximity. This figure shows box plots of the distribution of the log distance to the nearest gBGC tract, stratified by log distance to nearest telomere (first column) and recombination rate (second column) for both human (first row) and chimp (second row). For both species we observe that gBGC tracts are closer together towards the end of chromosomes (panels A and C), and that they are further apart in areas of low recombination rate (panels B and D). These empirical observations agree with the results of our linear modeling analysis (Text S1). (PDF) [file pgen.1003684.s004.pdf]

**A**

Distance to Nearest Tract (log)

Human

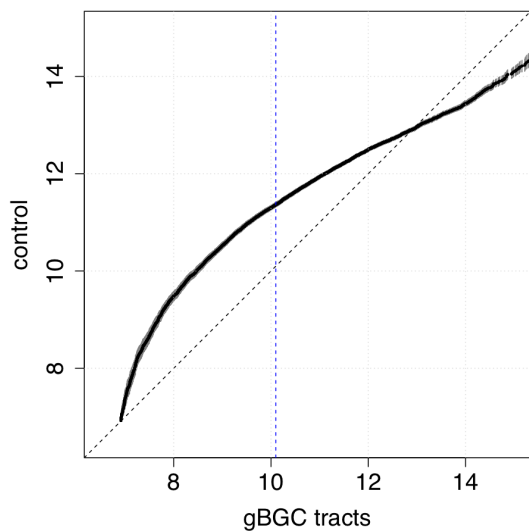**B**

Distance to Nearest Telomere (log)

control

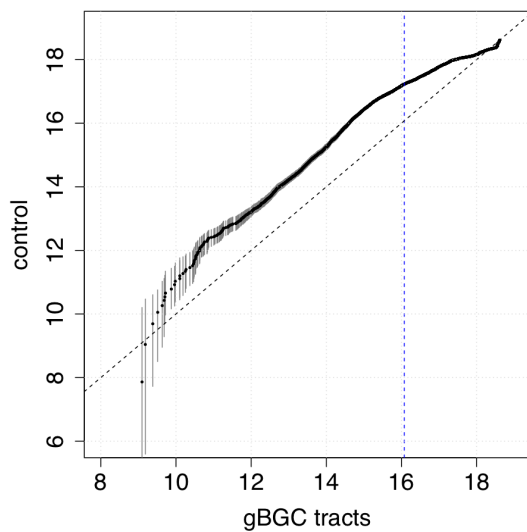**C**

Chimpanzee

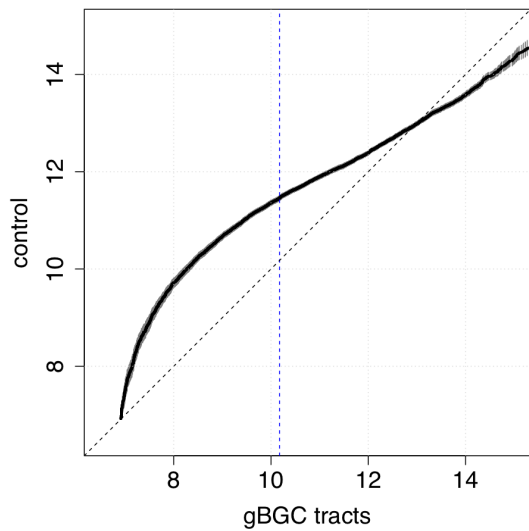**D**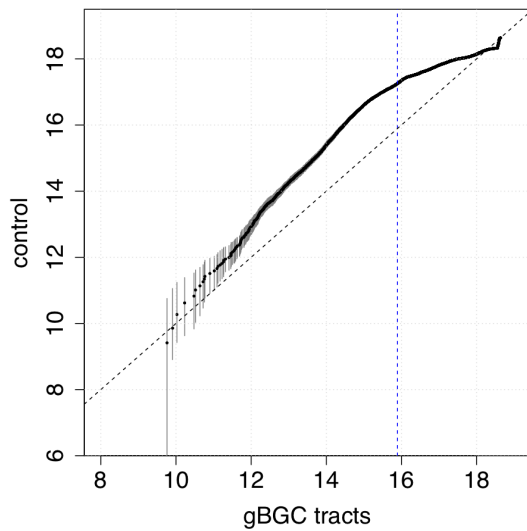

Supplement: Figure S5 — gBGC tracts are clustered and closer to telomeres than expected by chance. This figure shows qq-plots contrasting quantiles observed in gBGC tracts (x-axis) with medians of quantiles observed across GC-matched control sets (points, y-axis). The gray regions correspond to the data range observed across control regions (with the 1% highest and 1% lowest values removed). The vertical blue dashed line denotes the median for the gBGC tracts. Panels A and B show these plots for distance to nearest gBGC tract and the distance to nearest telomere in human; C and D show the corresponding plots for chimpanzee. (PDF) [file pgen.1003684.s005.pdf]

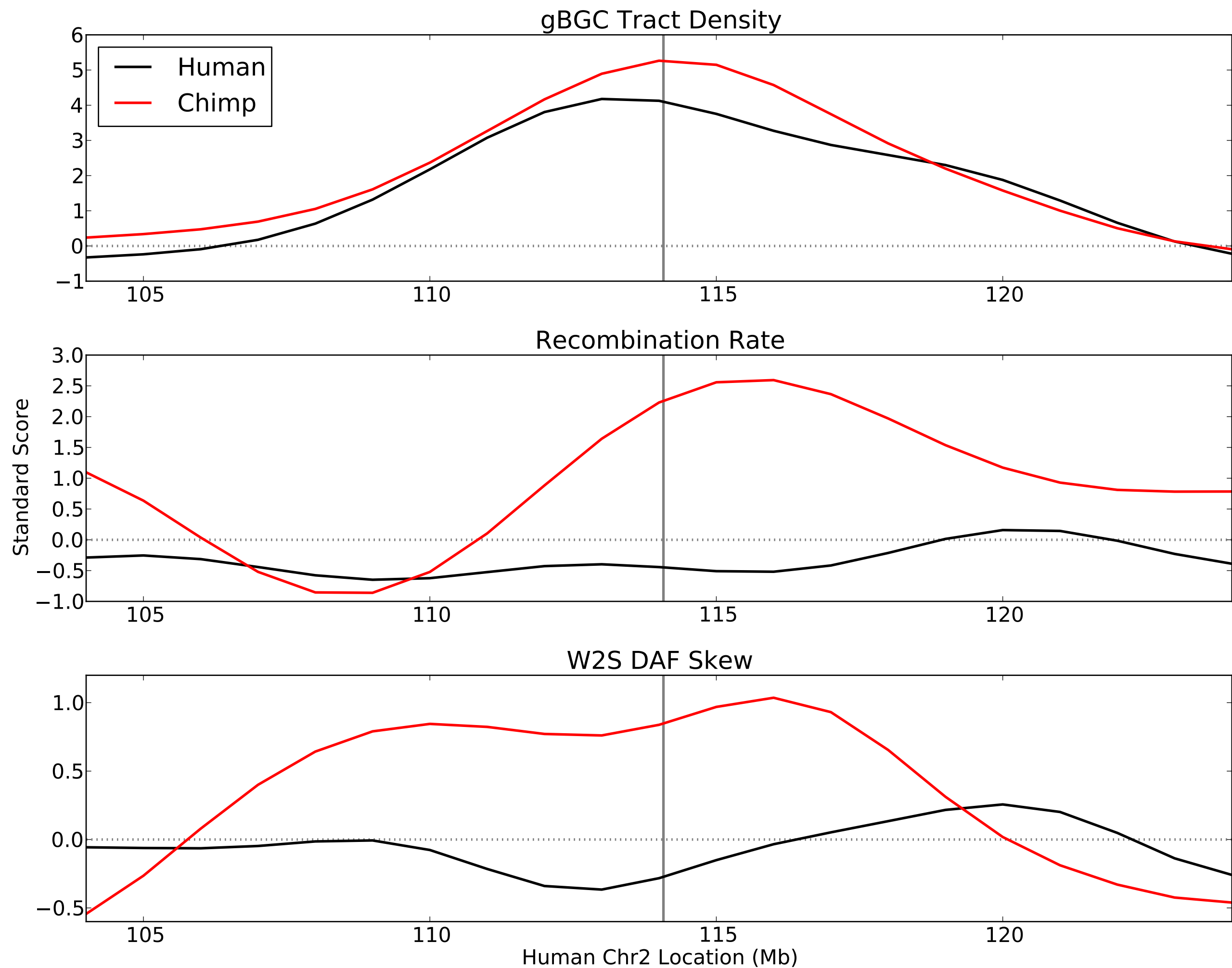

Supplement: Figure S6 — Signatures of recombination around the fusion site on human chromosome 2. Shown are predicted gBGC tract densities per megabase (top), crossover rates [25], [33] (middle), and DAF skews (bottom; see Methods) for a 20 Mb region centered on the fusion site on human chromosome 2 (gray vertical line). Separate lines represent data from the human genome (black) and the orthologous regions of chromosomes 2a and 2b in the chimpanzee genome (red). All measures are standardized by subtracting the chromosome-wide mean and dividing by the standard deviation. The raw data were smoothed using a Gaussian filter with . See the Discussion for interpretation of these differences between human and chimpanzee. (PDF) [file pgen.1003684.s006.pdf]

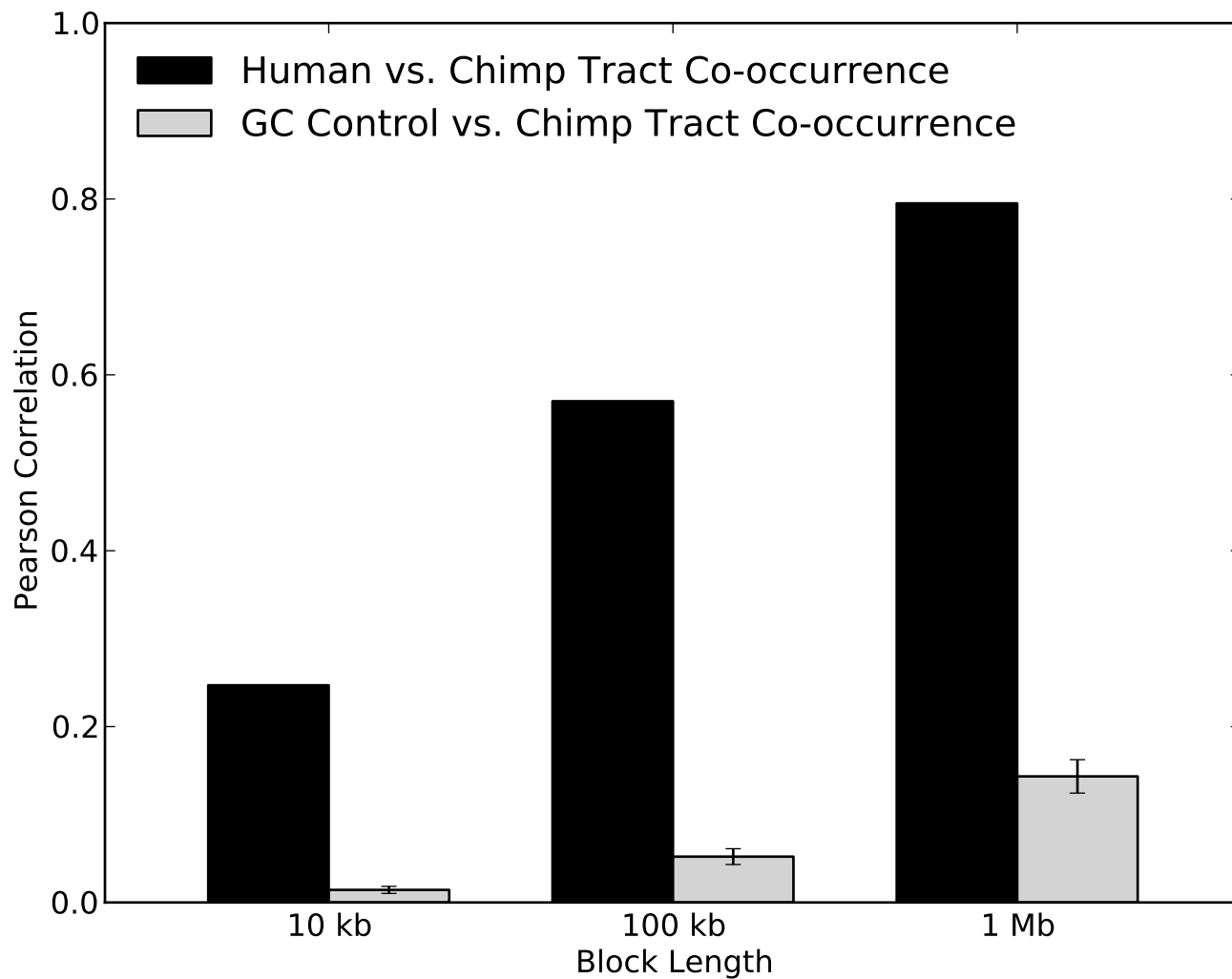

Supplement: Figure S7 — Human and chimpanzee gBGC tracts are found in broadly similar locations, but exhibit fine-scale differences. The fraction of bases in gBGC tracts is correlated between human and orthologous chimpanzee regions (Figure 4). The strength of this correlation increases as larger blocks of the genome are considered (x-axis). The gray bars give the average and standard deviation of the correlations observed between the gBGC fraction in 1000 GC-matched human control regions and the orthologous chimpanzee regions. (PDF) [file pgen.1003684.s007.pdf]

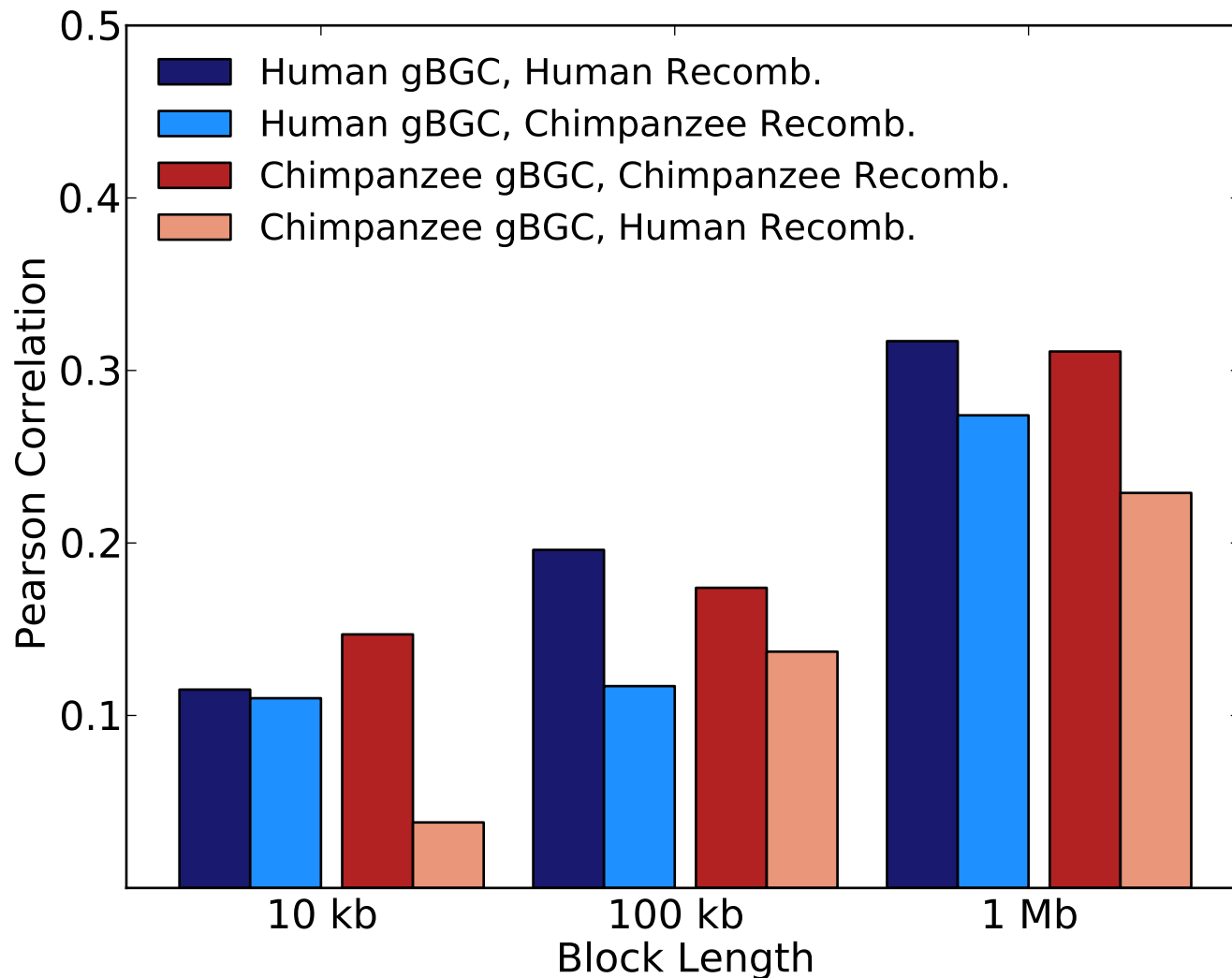

Supplement: Figure S8 — Correlation of recombination rates and gBGC tract densities within and between species. Recombination rates and gBGC densities are significantly correlated within species, and this correlation is more pronounced at larger scales (dark blue and dark red bars). When gBGC tract densities and recombination rates are compared across species (human gBGC tract densities vs.chimpanzee recombination rates or chimpanzee gBGC tract densities vs.human recombination rates; light blue and light red bars, respectively) they show weaker but still significant correlations. This plot considers only blocks that have nonzero values for all four statistics of interest. (PDF) [file pgen.1003684.s008.pdf]

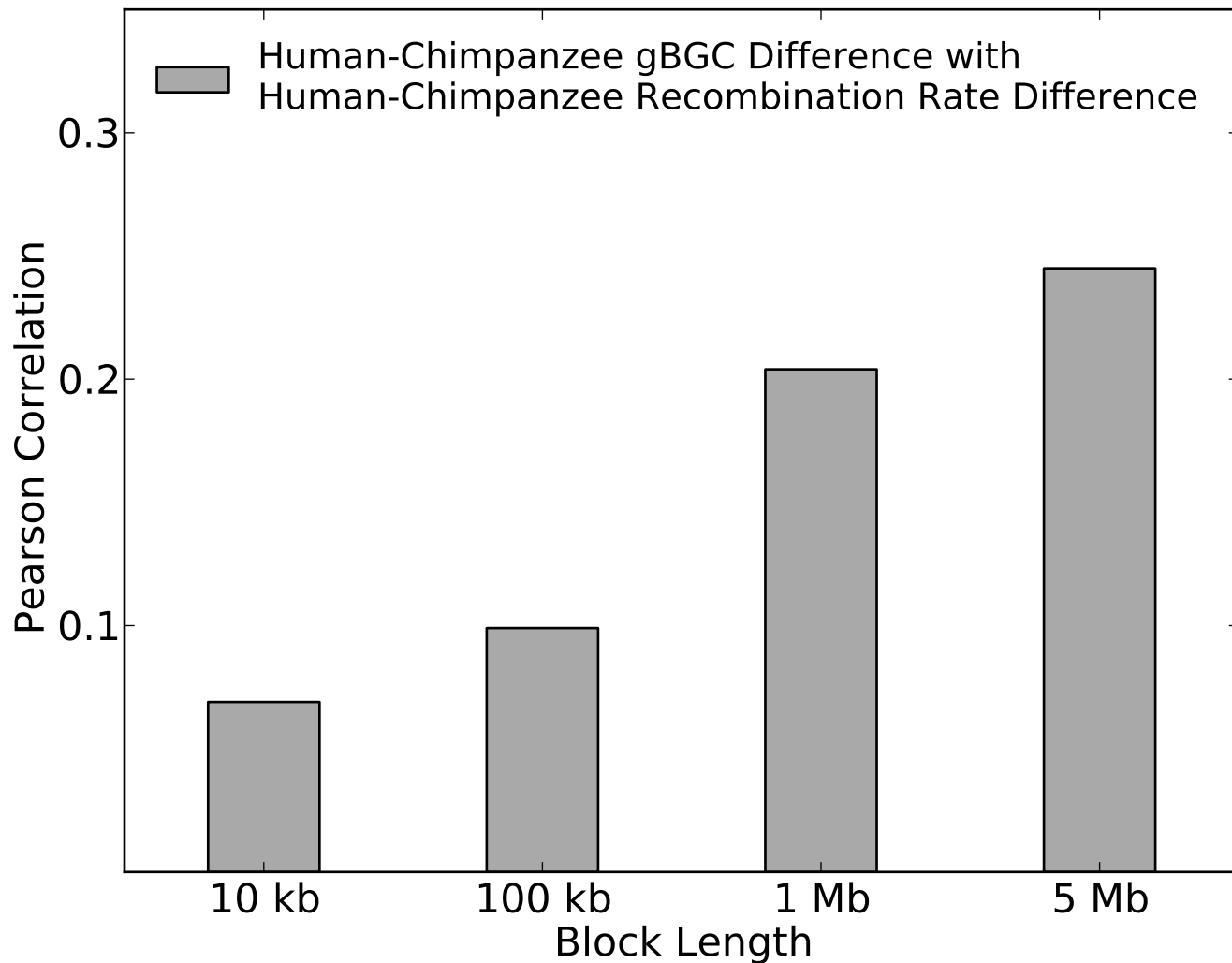

Supplement: Figure S9 — Differences in gBGC tract density between human and chimpanzee are modestly correlated with differences in recombination rate. Bars show the Pearson correlation between the difference between and , where and are the human and chimpanzee gBGC tract densities, respectively, and and are the human and chimpanzee recombination rates, respectively. Average values were computed for windows of various sizes (x-axis). All correlations are significantly greater than zero (10 kb: p = 0.01; 100 kb p = 6.5e–05; 1 Mb: p = 6.7e–11; 5 Mb: p = 1.7e–07). (PDF) [file pgen.1003684.s009.pdf]

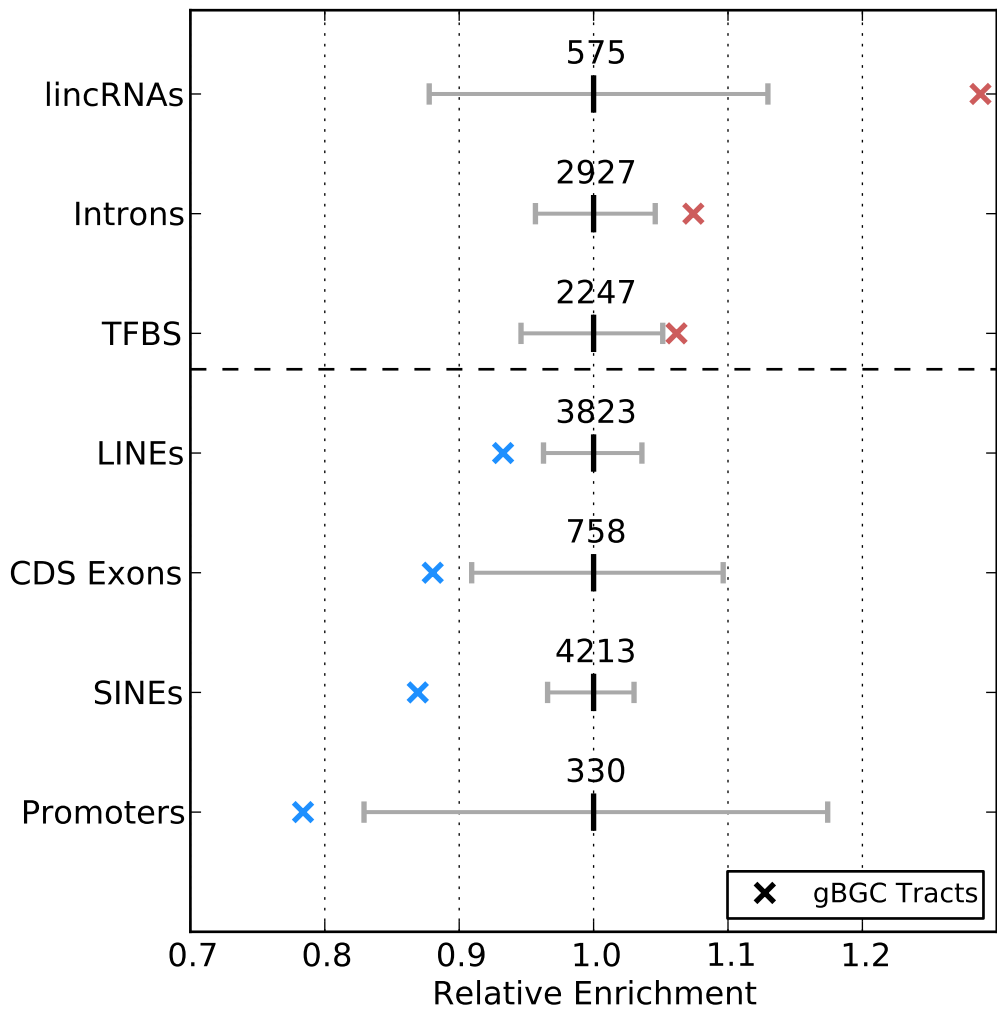

Supplement: Figure S10 — Genomic features significantly enriched or depeleted in gBGC tracts. For each genomic feature, we compared the number of overlaps observed with gBGC tracts with those observed in 1000 random GC-matched control regions. The gray bars give the minimum and maximum overlaps observed in the random sets. Shown are all features that are significantly () underrepresented (blue) or overrepresented (red) in the tracts. See the Methods for a full list of genomic features considered. Note that the tracts are more strongly enriched for recombination hotspots (not shown, 1.54×) and for high recombination rates (Table 3), both of which were considered separately. (PDF) [file pgen.1003684.s010.pdf]

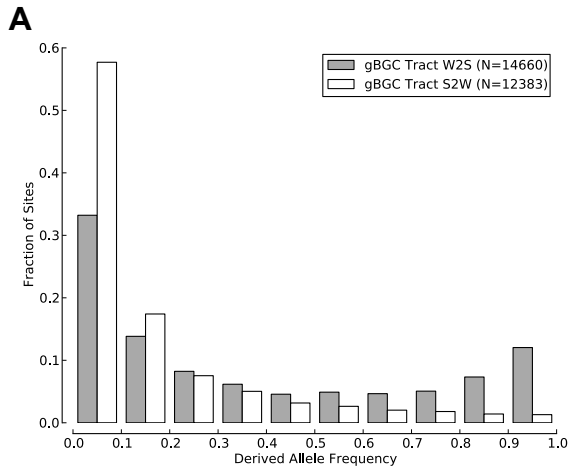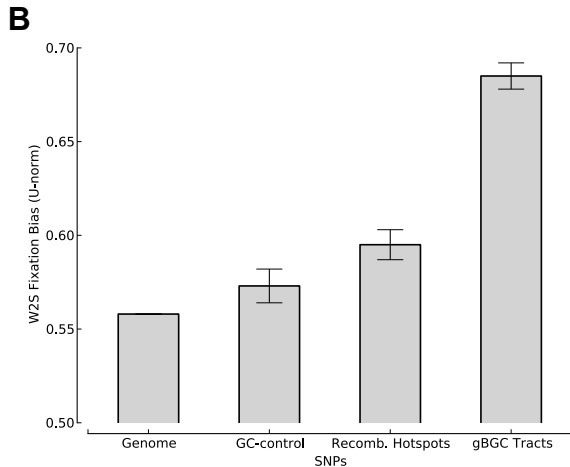

Supplement: Figure S11 — Human polymorphism data indicate an ongoing preference for the fixation of G and C alleles in the predicted gBGC tracts. This figure shows the same plots as Figure 5, but is based on an analysis in which polymorphic sites were masked from the alignments. (A) W→S changes in gBGC tracts have significantly higher derived allele frequencies than S→W changes. This result was obtained on the YRI population from the 1000 Genomes Project, and patterns for other populations were similar (data not shown). (B) The -norm, a measure of the degree of W→S bias (see Methods), is significantly higher in gBGC tracts than in the entire genome or in GC-matched control regions. Recombination hotspots also show somewhat elevated values but much less elevated than the predicted tracts. The error bars indicate 95% confidence intervals. (PDF) [file pgen.1003684.s011.pdf]

**A**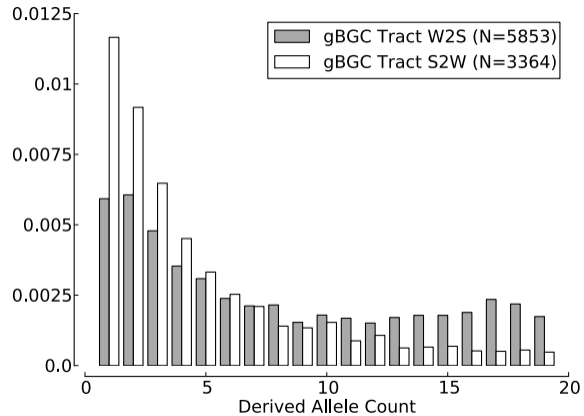**B**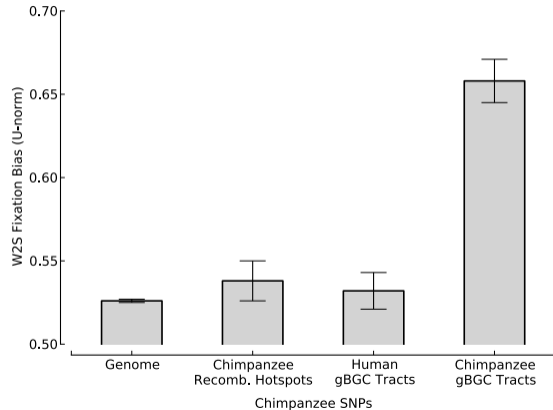

Supplement: Figure S12 — Chimpanzee polymorphism data indicate an ongoing preference for the fixation of G and C alleles in the predicted chimpanzee gBGC tracts. This figure shows the same analysis as Figure 5, but is based on chimpanzee polymorphism data for 10 individuals (20 chromsomes per site) from the PanMap project. (A) W→S changes in chimpanzee gBGC tracts have significantly higher derived allele frequencies than S→W changes. (B) Echoing the bias patterns observed in human polymorphism, the -norm, a measure of the degree of W→S bias (see Methods), is significantly higher in chimpanzee gBGC tracts than in the entire genome and human gBGC tracts mapped to the chimp genome. Chimpanzee recombination hotspots also show somewhat elevated values but much less elevated than the predicted tracts. The error bars indicate 95% confidence intervals. (PDF) [file pgen.1003684.s012.pdf]

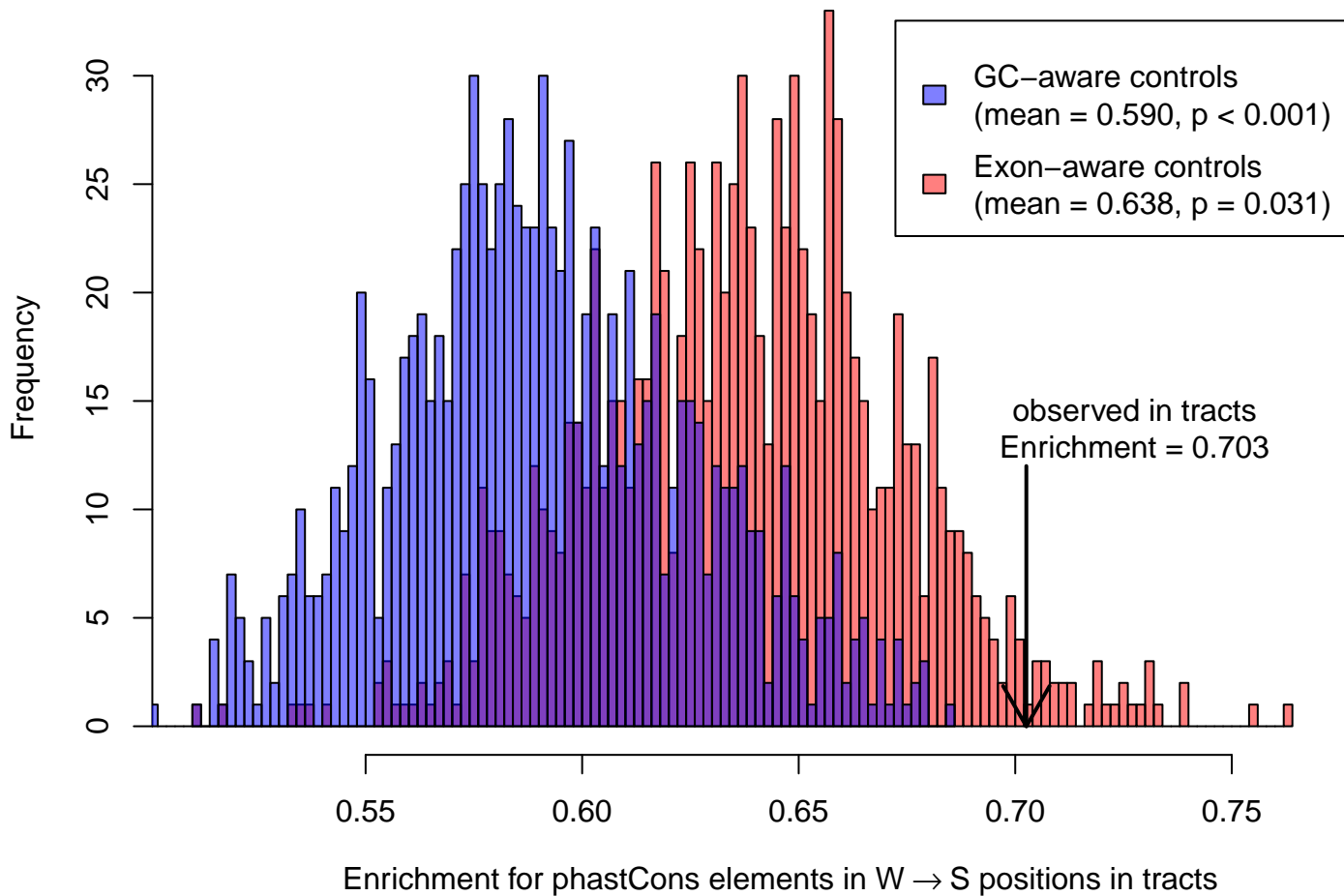

Supplement: Figure S13 — W→S sites within the predicted human tracts are enriched for phastCons elements compared to controls. Enrichments were calculated as the number of W→S substitutions within tracts falling in phastCons elements, divided by the number expected if these were distributed independently. The histograms show enrichment in our sets of 1000 GC- and exon-aware control tracts, and the arrow shows the value observed in the gBGC tracts. (PDF) [file pgen.1003684.s013.pdf]

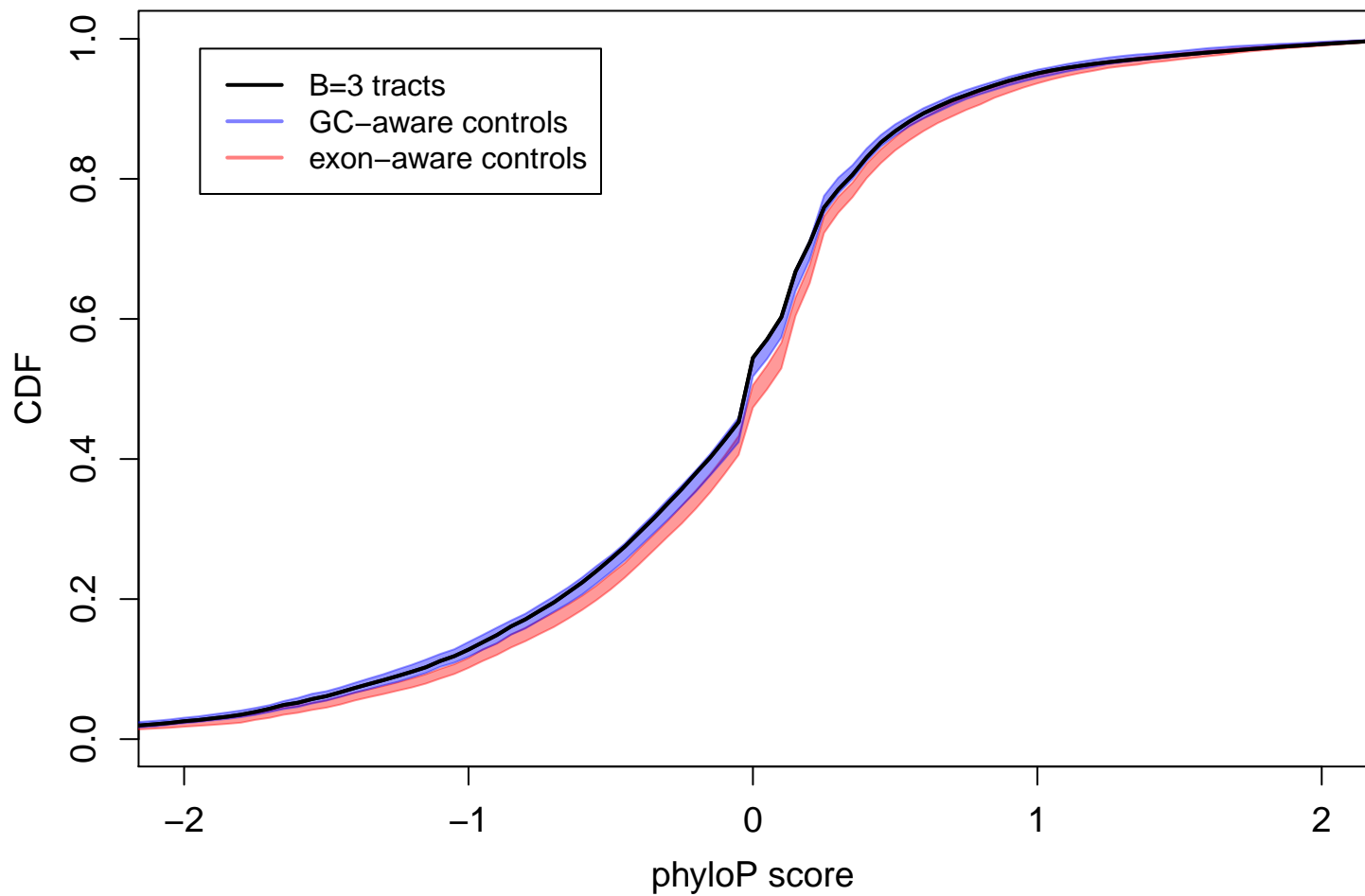

Supplement: Figure S14 — Conservation at sites of W→S substitutions within tracts. PhyloP scores were calculated at sites within the predicted human tracts at which W→S substitutions occurred on the human lineage. They were also calculated at sites of similar human-specific W→S substitution within the GC- and exon-matched control groups (1000 replicates). The scores were calculated for mammalian alignments from which the human and chimpanzee sequences had been removed. A higher phyloP score (x-axis) indicates greater evolutionary conservation. Although there are slight differences between the distributions for the tracts and the control groups, there is no clear excess of conservation at W→S sites in the tracts. (PDF) [file pgen.1003684.s014.pdf]

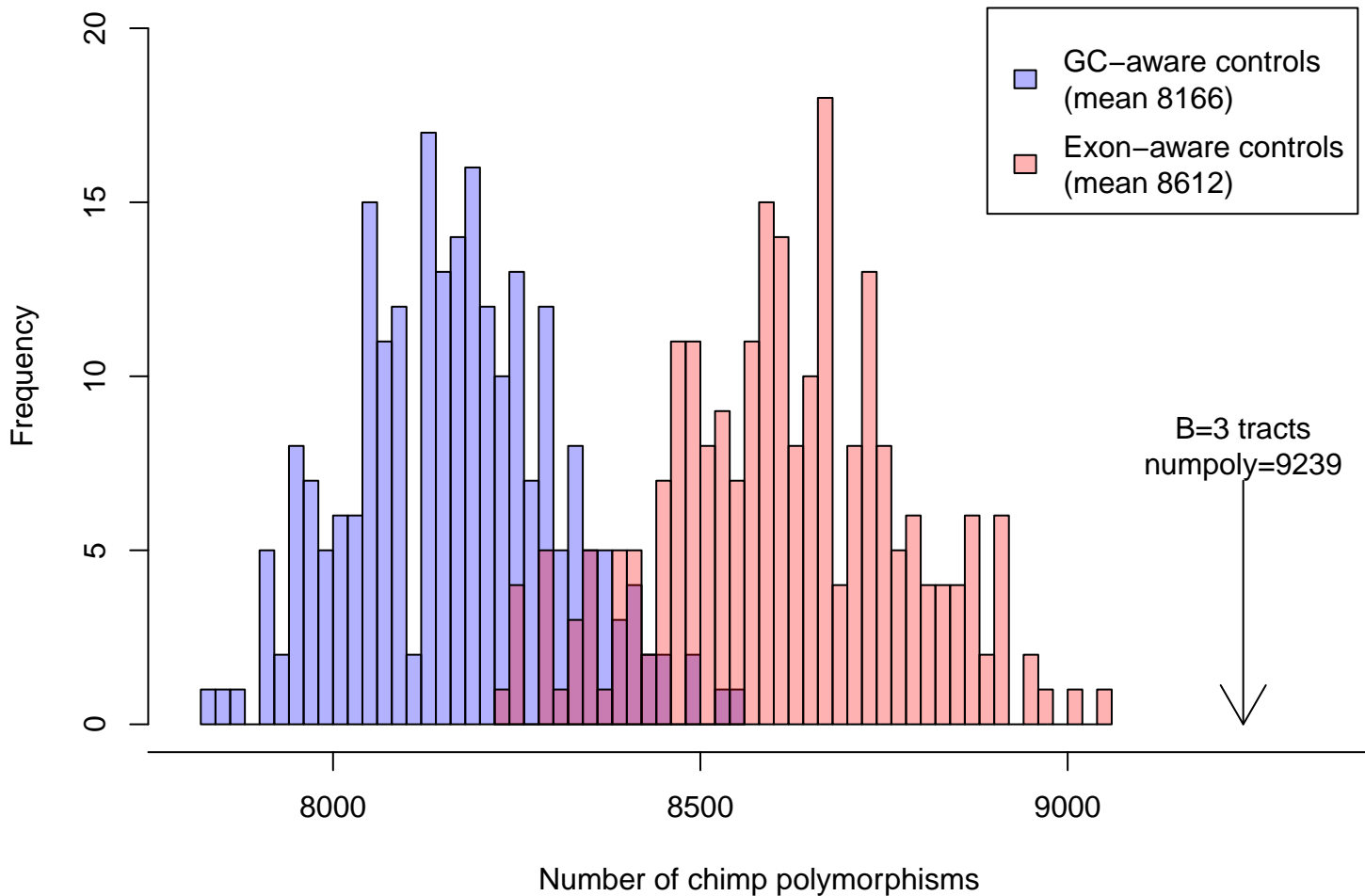

Supplement: Figure S15 — Number of chimpanzee polymorphisms in regions orthologous to the gBGC tracts, compared to controls. We observed significantly more chimpanzee polymorphisms in regions orthologous to the tracts than those orthologous to the control groups. This is the opposite of the observation that would be expected if the regions orthologous to the tracts were under purifying selection in the chimpanzee. (PDF) [file pgen.1003684.s015.pdf]

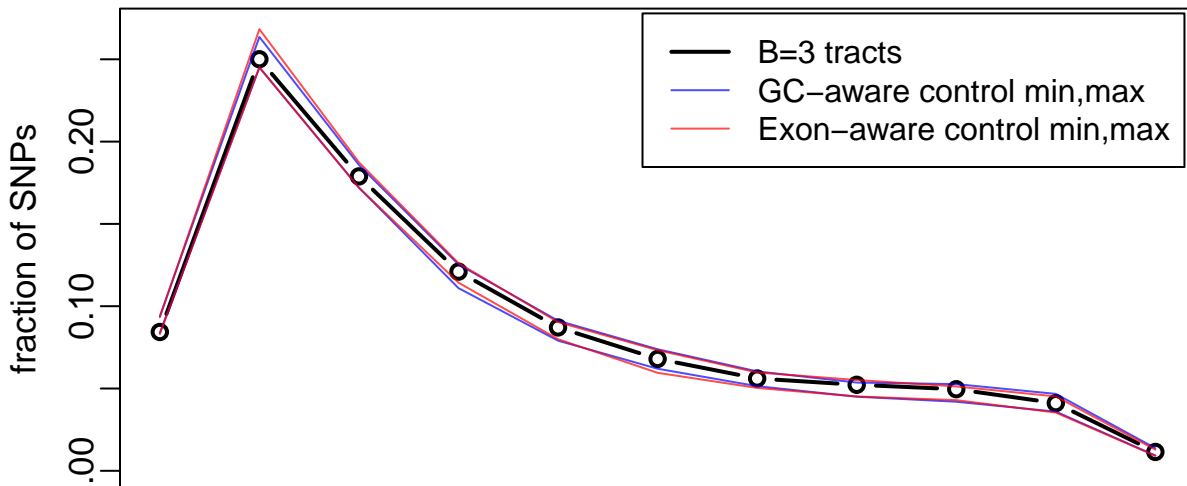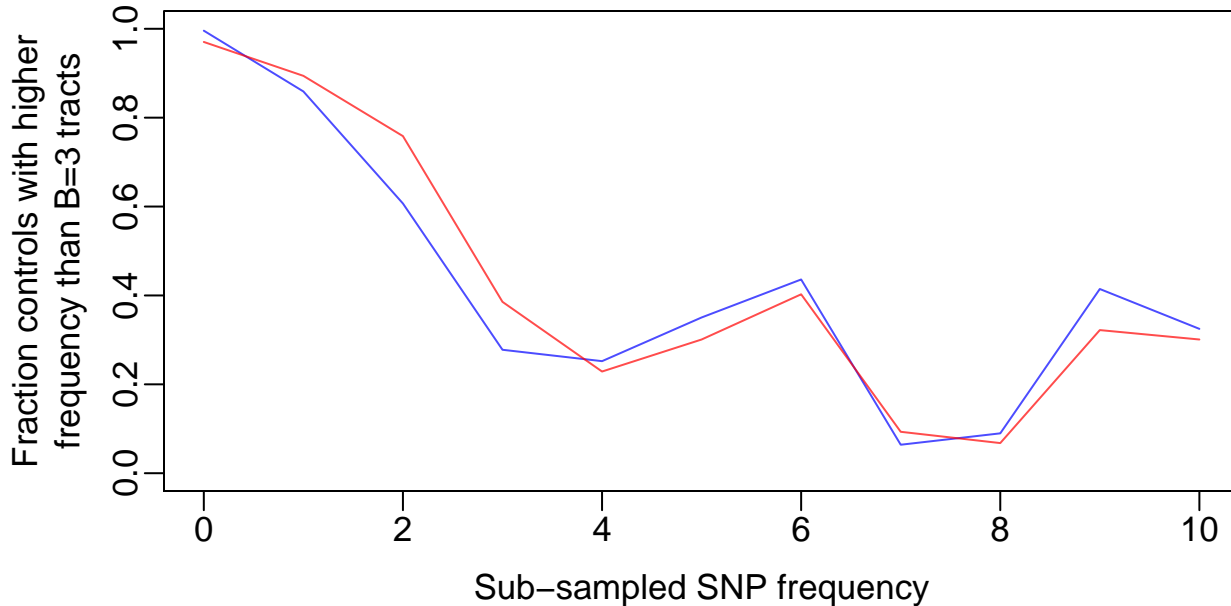

Supplement: Figure S16 — Derived allele frequency spectrum of chimpanzee polymorphisms in regions orthologous to the tracts. The top plot shows the derived allele frequency spectrum (polarized using the orangutan allele) for chimpanzee polymorphisms in regions orthologous to the gBGC tracts, compared with the minimum and maximum from 1000 control groups. The bottom plot shows the fraction of samples from each control group with a higher frequency than observed in the real tracts. We observe no significant excess of low-frequency derived alleles in regions orthologous to the tracts. (PDF) [file pgen.1003684.s016.pdf]
