# Supplementary material for: A Model-Based Analysis of GC-Biased Gene Conversion in the Human and Chimpanzee Genomes
Source: PLoS Genet. 2013 Aug 15;9(8):e1003684. doi: 10.1371/journal.pgen.1003684 (PMC3744432; doi:10.1371/journal.pgen.1003684)
Supplement: Table S1 — Relative coverage of human gBGC tracts for various values of B. Each value in the table represents the fraction of nucleotides in the human gBGC tract predictions for the value of indicated for the row that also fall in the predictions for the value of indicated for the column. The numbers on the main diagonal are one by definition. The numbers above the main diagonal indicate the coverage of smaller sets (higher ) by larger sets (lower ), while the numbers below the main diagonal indicate the coverage of larger sets by smaller sets. (PDF) [file pgen.1003684.s017.pdf]

	***B*=2**	***B*=3**	***B*=4**	***B*=5**	***B*=10**
***B*=2**	1	0.97	0.95	0.93	0.88
***B*=3**	0.29	1	0.98	0.97	0.92
***B*=4**	0.19	0.64	1	0.99	0.94
***B*=5**	0.13	0.46	0.72	1	0.95
***B*=10**	0.06	0.20	0.31	0.44	1

Table S1: **Relative Coverage of Human gBGC Tracts for Various Values of *B*.** Each value in the table represents the fraction of nucleotides in the human gBGC tract predictions for the value of B indicated for the row that also fall in the predictions for the value of B indicated for the column. The numbers on the main diagonal are one by definition. The numbers above the main diagonal indicate the coverage of smaller sets (higher B) by larger sets (lower B), while the numbers below the main diagonal indicate the coverage of larger sets by smaller sets.
