# Supplementary material for: A Model-Based Analysis of GC-Biased Gene Conversion in the Human and Chimpanzee Genomes
Source: PLoS Genet. 2013 Aug 15;9(8):e1003684. doi: 10.1371/journal.pgen.1003684 (PMC3744432; doi:10.1371/journal.pgen.1003684)
Supplement: Table S2 — Relative coverage of tracts predicted using various block sizes. This table shows the robustness of tract predictions to the block size used in analysis. The tracts presented in the paper were computed in 10 Mb blocks (highlighted). As discussed in the Methods section, several free parameters were estimated separately for each block, including the rate into the gBGC state. In each row of this table is shown the fraction of bases predicted at a given block size which were also predicted using the block size indicated by the column header. Most pairs of block size choices have overlaps of greater than 80%, except for the case of 1 Mb blocks, where some overfitting appears to occur. Inspection of the predictions indicates that most differences between predictions are due to short tracts for which the posterior probability for gBGC is near the threshold of 50%. (PDF) [file pgen.1003684.s018.pdf]

	1 Mb	5 Mb	10 Mb	20 Mb	30 Mb
**1 Mb**	1.00	0.79	0.75	0.68	0.68
**5 Mb**	0.89	1.00	0.89	0.80	0.80
**10 Mb**	0.86	0.90	1.00	0.85	0.84
**20 Mb**	0.84	0.88	0.92	1.00	0.93
**30 Mb**	0.83	0.86	0.89	0.92	1.00

Table S2: **Relative coverage of tracts predicted using various block sizes** This table shows the robustness of tract predictions to the block size used in analysis. The tracts presented in the paper were computed in 10 Mb blocks (highlighted). As discussed in the Methods section, several free parameters were estimated separately for each block, including the rate into the gBGC state. In each row of this table is shown the fraction of bases predicted at a given block size which were also predicted using the block size indicated by the column header. Most pairs of block size choices have overlaps of $\geq 80\%$, except for the case of 1 Mb blocks, where some overfitting appears to occur. Inspection of the predictions indicates that most differences between predictions are due to short tracts for which the posterior probability for gBGC is near the threshold of 50%.
