# Supplementary material for: A Model-Based Analysis of GC-Biased Gene Conversion in the Human and Chimpanzee Genomes
Source: PLoS Genet. 2013 Aug 15;9(8):e1003684. doi: 10.1371/journal.pgen.1003684 (PMC3744432; doi:10.1371/journal.pgen.1003684)
Supplement: Text S1 — Text describing additional analyses in support of the manuscript. (PDF) [file pgen.1003684.s019.pdf]

A Model-Based Analysis of GC-Biased Gene Conversion in the Human and Chimpanzee Genomes

Supplementary Information: Text S1

John A. Capra[1,a,†], Melissa J. Hubisz[2,†], Dennis Kostka[3], Katherine S. Pollard[1,4,*], and Adam Siepel[2,*]

[1]Gladstone Institutes, University of California, San Francisco, CA 94158, USA
[2]Dept. of Biological Statistics and Computational Biology, Cornell University, Ithaca, NY 14853, USA
[3]Depts. of Developmental Biology and Computational & Systems Biology, University of Pittsburgh, Pittsburgh, PA 15201 USA
[4]Institute for Human Genetics and Division of Biostatistics, University of California, San Francisco, CA 94107, USA
[a]*Current Address:* Dept. of Biomedical Informatics and Center for Human Genetics Research, Vanderbilt University, Nashville, TN 37232 USA
[†]These authors contributed equally to this work.
[*]To whom correspondence should be addressed; E-mail: acs4@cornell.edu; kpollard@gladstone.ucsf.edu.

Emission probabilities for phylo-HMM

In this section, we briefly describe the computation of emission probabilities for our phylo-HMM. Additional information can be found in references [1, 2].

Each state $j \in (N_0, N_B, C_0, C_B)$ in the phylo-HMM has a phylogenetic model ψ_j associated with it which is used to calculate the emissions probabilities for each alignment column. The phylogenetic models consist of:

- $\boldsymbol{\tau}$: the topology of the phylogenetic tree
- $\boldsymbol{\pi}$: the equilibrium frequencies of the four nucleotide bases
- $\boldsymbol{\beta}_j$: the branch lengths of the tree
- $\mathbf{Q}^{(f)}$: the substitution rate matrix used for the foreground branch of the tree
- $\mathbf{Q}^{(b)}$: the substitution rate matrix used for the background branches

In our case, the parameters $\boldsymbol{\tau}$ and $\boldsymbol{\pi}$ do not vary among the various HMM states. The topology $\boldsymbol{\tau}$ is assumed to be known, and $\boldsymbol{\pi}$ is pre-estimated using the program phyloFit, in a block-specific manner (see Methods).

The branch lengths $\boldsymbol{\beta}$ are also pre-computed, however for the conserved states (C_0 and C_B), all branch lengths are scaled by the conservation scaling parameter ρ, which was fixed at 0.31 in this study (Table 1).

For the non-gBGC states N_0 and C_0, the we use substitution rate matrices defined by the HKY85 model [3] for both the foreground and background branches:

$$Q^{(f)} = Q^{(b)} = \begin{pmatrix} * & \pi_C & \kappa\pi_G & \pi_T \\ \pi_A & * & \pi_G & \kappa\pi_T \\ \kappa\pi_A & \pi_C & * & \pi_T \\ \pi_A & \kappa\pi_C & \pi_A & * \end{pmatrix}$$

Here, the rows and columns of the matrix follow an ordering of (A, C, G, T) for the four nucleotides and the *'s along the main diagonal indicate values needed to satisfy the constraint that all rows must sum to zero. κ is the transition-transversion rate ratio, which, along with $\boldsymbol{\pi}$, is pre-estimated for each alignment block using phyloFit.

In the gBGC states, the background substitution matrices are the same as in the non-gBGC states. However the foreground substitution matrices are altered to model the effects of gBGC. As in Kostka et al. [4], the rate of W→S mutations is scaled by $\frac{B}{1-e^{-B}}$, whereas the rate of S→W mutations is scaled by $\frac{-B}{1-e^{B}}$. Therefore, for states N_B and C_B, we have:

$$Q^{(f)} = \begin{pmatrix} * & \frac{B}{1-e^{-B}}\pi_C & \frac{B}{1-e^{-B}}\kappa\pi_G & \pi_T \\ \frac{-B}{1-e^{B}}\pi_A & * & \pi_G & \frac{-B}{1-e^{B}}\kappa\pi_T \\ \frac{-B}{1-e^{B}}\kappa\pi_A & \pi_C & * & \frac{-B}{1-e^{B}}\pi_T \\ \pi_A & \frac{B}{1-e^{-B}}\kappa\pi_C & \frac{B}{1-e^{-B}}\pi_A & * \end{pmatrix}$$

For a given state, the emissions probabilities are calculated in the usual way for statistical phylogenetic models, after choosing the appropriate substitution matrix $\mathbf{Q}$ and branch length t for each branch. The probability of transition from any nucleotide to any other along each branch is calculated by taking the matrix exponential, e^{Qt}. Given these probabilities and the equilibrium frequencies $\boldsymbol{\pi}$, Felsenstein's pruning algorithm [5] is used to calculate $P(\mathbf{X}_i|\boldsymbol{\psi}_j)$ for each column $\mathbf{X}_i$ of the alignment. These columnwise likelihoods are the emission probabilities for the HMM.

Clustering, recombination rate, and distance to telomeres

To investigate the degree to which the predicted gBGC tracts are clustered, we calculated for each gBGC tract the distance to the nearest other gBGC tract (distance-to-nearest). One potential caveat with this type of analysis is that gBGC tracts were annotated by thresholding posterior probabilities in our HMM (see Methods), which might cause predictions to occur very close together simply because of fluctuations in posterior probability along the genome. Therefore, for these analyses, we merged gBGC tracts with their neighbors if the distance between them was less than 1 kb (the expected gBGC tract length).

gBGC tracts are close in human and chimpanzee. To assess the closeness of gBGC-tracts in human and chimpanzee we repeated the distance-to-nearest calculation for 1,000 random GC-matched control sets (in human and chimp separately), and then contrasted the distribution we observed in these controls with the distribution in the gBGC-tracts. Panels A and C of Figure S5 summarize the results in terms of quantile-quantile plots between the two distributions. This illustrates that gBGC-tracts are significantly closer together than regions in control sets. For human, the median distance-to-nearest is 24,305 bp, while the average of median nearest distances across control sets is 86,064 bp (with a standard deviation of 1,571.1). For chimp, the median distance-to-nearest in the gBGC tracts is 26,286 bp, and the average median distance across control sets is 94,882 bp (sd = 1,888.2). In both species, we never observe a median distance-to-nearest in the control sets as low as in the gBGC tracts (z-score based $p < 10^{-100}$).

gBGC tracts are close to telomeres in human and chimpanzee. To assess distance to telomeres we performed the same analysis, but used distance to the nearest telomere in place of distance to the nearest neighboring tract (Figure S5, B and D). The median distance-to-telomere in human and chimpanzee are 9,568,725 bp and 8,002,971 bp, while the averages across control sets are 30,393,137 bp (sd = 329,252.1) and 31,209,994 bp (sd = 370,981.5), respectively. In neither species do we observe a median distance in the control sets that is as low as in the gBGC tracts ($p < 10^{-100}$).

Distance-to-telomere and recombination rate partially, but not entirely, account for the closeness of gBGC tracts. Next we asked to what extent distance-to-telomere and recombination rate could be driving the observed proximity of gBGC tracts to one another. To address this question we fit the following linear model to the data for each species:

$$E[X] = a + \beta_1 d + \beta_2 r.$$

Here, X denotes the logarithm of the distance to the nearest neighboring gBGC tract, d is the log distance-to-telomere, and r is the mean recombination rate of a tract. We find that, in both species, both β_1 and β_2 are significantly different from zero (see Table below) and the model predicts gBGC tracts to be close together near the telomeres and far apart in areas of low recombination rate (also see Figure S4). This is also reflected in Spearman correlation coefficients between distance-to-nearest and distance-to-telomere (0.36 in human, 0.49 in chimp) and recombination rate (-0.18 in human, -0.20 in chimp). Despite these significant associations the multiple coefficient of determination (R^2) of the linear model is only about 13% in human and 22% in chimpanzee. That is, the majority of the variance in the distance-to-nearest observations remains unexplained when taking distance-to-telomere and recombination rate into account.

species	coefficient	value	std. error	p-value
human	β_1	0.431	0.190	$< 10^{-15}$
human	β_2	-0.006	0.002	0.006
chimp	β_1	0.613	0.141	$< 10^{-15}$
chimp	β_2	-0.022	0.007	$9.8 \cdot 10^{-4}$

Distance to telomere and recombination rate are significant factors in predicting proximity of gBGC tracts. This table shows the coefficients for a linear model in which the log distance-to-nearest gBGC tract was regressed on the log distance-to-telomere (β_1) and recombination rate (β_2). p-values are based on the T-test. The intercept is significant in both species.

Evidence of purifying selection in regions orthologous to gBGC tracts

We sought additional support for a link between gBGC and deleterious alleles by looking for evidence of purifying selection in chimpanzees and other species at the locations of W→S substitutions within the predicted human tracts. If a substantial number of these mutations were driven to fixation by gBGC despite negative selection against them, we would expect to observe an excess of evolutionary conservation, a deficiency of polymorphisms, and/or a skew toward low-frequency derived alleles at orthologous locations in other species. (This hypothesis assumes that gBGC tracts rarely occur at the same locations in multiple lineages, as suggested by the minimal overlap of our human and chimpanzee tracts.) However, we found that the bulk distributions of phyloP conservation scores [6], computed for eutherian mammals but excluding human and chimpanzee (see below), were nearly identical for the tracts and the GC-matched controls (Figure S14). In addition, we compared chimpanzee polymorphisms [7] in regions orthologous to our human tracts and control regions, and found no deficiency of polymorphisms (Figure S15) and no excess of low-frequency derived alleles (Figure S16) within the tracts. Indeed, the regions orthologous to the human tracts

displayed an excess of chimp polymorphisms, perhaps reflecting increased power for gBGC detection in regions of elevated mutation rates or the moderately increased chimp recombination rate in these regions. On the other hand, we did observe a significant enrichment for overlap with evolutionarily conserved elements identified using phastCons [8] at locations of W→S substitutions within the predicted tracts (Figure S13). Thus, this line of analysis yielded mixed evidence linking gBGC with fixation of deleterious alleles.

Methods for purifying selection analyses. We evaluated evidence of purifying selection at regions of other mammalian genomes by considering (1) evolutionary conservation scores for mammals and (2) patterns of polymorphism in chimpanzee. In both cases, we compared regions orthologous to the predicted human tracts and regions orthologous to control regions. The first comparison used phyloP CONACC conservation scores [6] at positions of human-specific W→S substitutions within the predicted tracts. To avoid possible biases from human-specific substitutions or from the chimpanzee sequence used to assign these substitutions to the human branch, we re-computed the phyloP scores based on alignments of eutherian mammals from which the human and chimpanzee sequences had been removed (leaving 30 mammalian species). We compared the tracts with both exon- and GC-matched control regions (Figure S14). For the polymorphism analysis, we used data from the PanMap Project [7] and considered all nucleotides within the predicted tracts, because the subset of sites with both human-specific W→S substitutions and chimpanzee polymorphisms was very small (42 sites).

References

1. Siepel A, Haussler D (2004) Combining phylogenetic and hidden Markov models in biosequence analysis. J Comput Biol 11: 413-428.

2. Siepel A, Haussler D (2005) Phylogenetic hidden Markov models. In: Nielsen R, editor, Statistical Methods in Molecular Evolution, New York: Springer. pp. 325-351.

3. Hasegawa M, Kishino H, Yano T (1985) Dating the human-ape splitting by a molecular clock of mitochondrial DNA. J Mol Evol 22: 160-174.

4. Kostka D, Hubisz MJ, Siepel A, Pollard KS (2012) The role of GC-biased gene conversion in shaping the fastest evolving regions of the human genome. Mol Biol Evol 29: 1047–1057.

5. Felsenstein J (1981) Evolutionary trees from DNA sequences: a maximum likelihood approach. J Mol Evol 17: 368-376.

6. Pollard KS, Hubisz MJ, Rosenbloom KR, Siepel A (2010) Detection of nonneutral substitution rates on mammalian phylogenies. Genome Res 20: 110–121.

7. Auton A, Fledel-Alon A, Pfeifer S, Venn O, Segurel L, et al. (2012) A fine-scale chimpanzee genetic map from population sequencing. Science 336: 193-198.

8. Siepel A, Bejerano G, Pedersen JS, Hinrichs AS, Hou M, et al. (2005) Evolutionarily conserved elements in vertebrate, insect, worm, and yeast genomes. Genome Res 15: 1034–1050.
